# Effectiveness of menstruation and fertility tracking technology in childbearing women: a scoping review

**DOI:** 10.1590/1980-220X-REEUSP-2024-0454en

**Published:** 2025-10-13

**Authors:** Yi Li, Yuhui Fang, Shuiqin Gu

**Affiliations:** 1 Zhejiang Chinese Medical University, Master Degree Cultivation Base, Zhejiang Province, China.; 2 Jiaxing University, Jiaxing Maternity and Children Health Care Hospital, Nursing Department, Jiaxing, Zhejiang Province, China.

**Keywords:** Ovulation Detection, Fertility Window, Menstruation, Pregnancy, Scoping Review, Detecção da Ovulação, Janela Fértil, Menstruação, Gravidez, Revisão de Escopo

## Abstract

**Objective:**

To investigate standardized temperature measurement protocols and intelligent data processing methods to improve ovulation prediction accuracy.

**Method:**

Based on Arksey and O'Malley's scoping review reporting framework, relevant publications from August 15, 2014, to August 15, 2024, were retrieved from the MEDLINE, EMBASE, SCOPUS, and Web of Science databases. The publications were screened, summarized, and evaluated according to the Critical Appraisal Skills Programme to assess their rigor.

**Results:**

A total of 21 publications reporting studies from 9 countries involving 26,044 participants were included. Fertility tracking system measurement devices based on basal body temperature (BBT) included wearable devices and basal body thermometers. The application functions included menstrual assessment, fertility prediction, contraception, and pregnancy management. The applications were evaluated in terms of functionality and user experience.

**Conclusion:**

Research into the application of fertility tracking based on BBT remains in the preliminary stage. The findings of this study provide a valuable reference for the development of personalized and convenient applications, which requires high-quality prospective cohort research.

## RESUMO

**Objetivo:**

Investigar protocolos padronizados de medição de temperatura e métodos inteligentes de processamento de dados para melhorar a precisão da previsão da ovulação.

**Método:**

Com base no arcabouço de relato de revisão de alcance de Arksey e O’Malley, foram recuperadas publicações relevantes de 15 de agosto de 2014 a 15 de agosto de 2024 nas bases de dados MEDLINE, EMBASE, SCOPUS e Web of Science. As publicações foram selecionadas, resumidas e avaliadas de acordo com o Programa de Habilidades de Avaliação Crítica (CASP) para avaliar seu rigor.

**Resultados:**

Foram incluídas um total de 21 publicações que relatavam estudos realizados em 9 países e envolveram 26.044 participantes. Os dispositivos de medição de sistemas de acompanhamento da fertilidade baseados na temperatura corporal basal (TCB) incluíram dispositivos portáteis e termômetros de temperatura corporal basal. As funções das aplicações incluíram avaliação menstrual, previsão de fertilidade, anticoncepção e gestão do embarazo. As aplicações foram avaliadas em termos de funcionalidade e experiência do usuário.

**Conclusão:**

As pesquisas sobre a aplicação do acompanhamento da fertilidade baseado na TCB ainda estão em estágio preliminar. Os achados deste estudo fornecem uma referência valiosa para o desenvolvimento de aplicações personalizadas e convenientes, o que requer pesquisas prospectivas de coorte de alta qualidade.

## INTRODUCTION

Basal body temperature (BBT) can be used as a sign of fertility and is commonly utilized to estimate the fertility window through consistent monitoring, including digital tracking in health applications (apps)^(1)^. The principle behind monitoring is that BBT presents a biphasic pattern due to the thermogenic effect of progesterone, a hormone that increases after ovulation^(2)^. Currently, approximately 68% of fertility tracking applications rely on self-reporting BBT and menstrual cycle data to predict menstrual tracking and fertility, as it is a cost-effective, easy-to-use, and non-invasive method^(3,4)^. These applications often encompass various functions, such as tracking menstrual cycles, recording symptoms, contraception and family planning, and pregnancy monitoring^(5)^. However, despite their popularity, these applications have been criticized for potential harm due to concerns about their accuracy and the lack of robust evidence supporting their efficacy, which may lead to potential risks such as unintended pregnancies or delayed diagnosis of infertility^(6,7)^. Therefore, there is an urgent need to enhance the predictive accuracy of these applications and strengthen the relevant evidence.

With the development of wearable devices and advancements in machine learning algorithms, precise prediction of the fertility window is becoming feasible. When worn at night on various parts of the body, such as the wrist or distal areas, wearable devices can provide continuous and detailed skin temperature data^(8,9)^. Multiple studies have confirmed the consistency between nighttime skin temperature and BBT^(8,10)^. At the same time, innovative machine-learning algorithms have been developed to analyze extensive time-series data to improve the accuracy of ovulation day prediction^(8,11)^.

Despite these advances, no systematic review has evaluated the current status of measurement devices, the function of intelligent algorithms, or the effectiveness of fertility tracking applications based on BBT. To address this gap, a scoping review was proposed that could identify research progress in specific thematic areas more rapidly than traditional reviews while providing an overview that could drive updates in knowledge systems within this field^(12)^. Based on this proposition, the present study aimed to conduct a systematic scoping review based on Arksey and O’Malley’s scoping review framework to evaluate the accuracy, functionalities, and user experience of fertility tracking applications that use intelligent algorithms and are based on BBT. The findings provide a reference and foundation for future research on fertility tracking applications based on BBT among women of childbearing age.

## METHOD

### Study Design

This scoping review followed the methodological framework of the Joanna Briggs Institute (JBI), which aims to provide answers to a well-defined research question. Including research of various designs, this framework describes the extent, range, and nature of research and identifies lacunae in the existing literature. It consists of five steps: scoping, searching, screening, data extraction, and data analysis^(13)^. Reporting of the methods and findings was guided by the Preferred Reporting Items for Systematic Reviews and Meta-Analysis (PRISMA) criteria^(14)^. Data for this review were publicly available, so approval by the institution’s ethics committee was unnecessary.

### Research Questions

This scoping review addresses the following research questions: (1) What are the monitoring devices for BBT, and how can confounding factors be controlled? (2) What is the specific content of menstrual and fertility tracking applications? (3) What are the effects of applying menstrual and fertility tracking applications?

### Research Strategy

The eligibility of the studies was assessed based on the population, concept, and context (PCC) framework suggested by the JBI^(15,16)^. Table 1 provides an overview of the PCC criteria and the types of evidence considered in this scoping review. The MEDLINE, EMBASE, SCOPUS, and Web of Science databases were searched for all English-language articles published between August 15, 2014, and August 15, 2024. The detailed strategy on PubMed was use of the following Medical Subject Headings (MeSH): ((women AND (humans[Filter]) AND (“Basal Body Temperature”[Title/Abstract] OR “BBT”[Title/Abstract] OR “Skin Temperature”[Title/Abstract] OR “WST”[Title/Abstract] OR “core body temperature”[Title/Abstract] OR “thermal method”[Title/Abstract] OR “Symptothermal method”[Title/Abstract] OR “temperature Sensor”[Title/Abstract]) AND (“system*”[Title/Abstract] OR “device”[Title/Abstract] OR “app*”[Title/Abstract] OR “computers”[MeSH])) AND (2014:2024[pdat])). Another database search strategy that was devised by investigators and complied with the Peer Review of Electronic Search Strategies checklist was also used (Additional File 1).

**Table 1:** Inclusion and exclusion criteria based on the population, concept, and context framework.

	Inclusion criteria	Exclusion criteria
Population	Women of childbearing age	Pregnant or lactating women
Concept	Tracking applications for predicting menstruation and fertility window	Focus only on the user experience and usability of the application
Context	Different menstruation and fertility tracking system applications based on basal body temperature	Not applicable
Types of Evidence	RCTs; quasi-RCTs; and qualitative, quantitative, and mixed methods studies	Reviews, meta-analyses, animal experiments, and articles without full text

RCTs: randomized controlled trials.

### Data Extraction

Concurrent with screening, a data extraction table was developed to collect information on each study’s authors, publication year, country, research design, research objective, method of measuring BBT, confounding factors, data processing and modeling, and application usage and effectiveness. Two investigators independently verified the accuracy of the data in the table.

### Study Screening

Three investigators establish a shared understanding of the content and effectiveness of the menstrual and fertility tracking applications. Two investigators independently screened the titles and abstracts of 1357 records and compared and discussed the findings of the selected articles that met the inclusion criteria, which were considered eligible for full-text screening. The investigators sought advice from a third person in case of disagreement.

### Quality Appraisal

Quality analysis of the literature included in the scoping review was performed to assess its strengths and limitations. The 2024 Critical Appraisal Skills Programme (CASP) tool was applied to facilitate a systematic assessment of the key methodological components, including study design, validity, rigor, and relevance to the research question^(17)^. Two investigators independently performed the quality assessment and subsequently reached a consensus regarding the methodological quality of the studies. The assessment classified the studies into three categories: no concerns (answered “Yes” to all criteria), minor concerns (answered “Yes” to all except one criterion, to which was answered “No” or “Can’t Tell”), and major concerns (answered “No” or “Can’t Tell” to more than one criterion). Any disagreements were resolved through discussion.

### Data Analysis

Data synthesis and analysis were conducted by two reviewers after they reached consensus through discussion. Initial categories were developed through eventual agreement, with the extracted data examined, discussed, compared, and collated. Categories were refined and adjusted through discussion and re-assessment of the extracted data, with final categories named and presented in a tabular format.

## RESULTS

### Search Results

**Figure 1:**
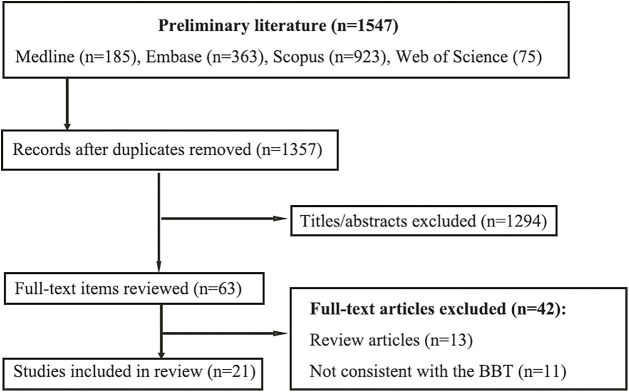
PRISMA study selection flowchart.

A total of 1547 studies were initially identified, and 190 were removed as duplicates. Review of the titles and abstracts excluded 1294 articles, leaving 63 full-text articles to be screened. Of these, 42 articles were excluded; 13 because they were review articles, 11 because they were not consistent with the BBT’s definition, and 18 because they did not report tracking applications, leaving a total of 21 studies eligible for review (Figure 1). The details of these studies are shown in Table 2.

**Table 2:** Data extracted from the 21 included studies.

Author	Publication year	Country	Research design	Research participants	Content and method of measurement	Confounding variables	Study data processing	Application functionality	Application effectiveness outcomes
Zhu et al.^(8)^	2021	Switzerland	Prospective cohort study	57 healthy women aged 18–45 years, included 193 cycles	1. Nocturnal wrist skin temperature: Ava Fertility Tracker bracelet, a wearable device 2. BBT: Lady-Comp, a digital thermometer 3. Reference standard for ovulation: ClearBlue Digital Ovulation Test, a home-based urine LH test	Age, weight, height, race, and time since stopping hormonal contraception	1. Data processing: Three-over-six rule 2. Modeling method: A linear mixed effects model	Ava app, a computerized fertility tracker 1. Data transmission: Anonymously synchronize data 2. Usage: Detect ovulation	Wrist skin temperature vs. BBT Detecting ovulation 1. Sensitivity 2. Specificity 3. Probability that ovulation was detected 4. Negative predictive values 5. Repeated measures correlation coefficient 6. Range of temperature increase
Yu et al.^(18)^	2022	China	Prospective cohort study	114 nonpregnant women aged 18–45 years, included 382 cycles	1. Heart rate: Huawei Band 5, a wearable device 2. BBT: Braun IRT6520, an ear thermometer 3. Reference standard for ovulation: Ovarian ultrasound and serum hormone levels	Age, weight, height, marital status, educational attainment, occupation, age at menarche, smoking status, and alcohol consumption	1. Data correction: Bonferroni method 2. Data analyses and visualizations: R software 3. Modeling method: Linear mixed model	Huawei app 1. Data transmission: Synchronize data 2. Usage: Detect fertile window and menses	Regular vs. irregular menstruators Fertile window and menses Accuracy Sensitivity Specificity Area under the curve
Scherwitzl et al.^(19)^	2015	Switzerland	Retrospective study	317 women aged 18–39 years, included 1501 cycles	1. BBT: Basal thermometer 2. Reference standard for ovulation: LH tests 3. Date of menstruation	Age, cycle length, cycle variation, sexual activity, pregnancy plans, pregnancy status, BMI, smoking status, past use of hormonal contraception	1. Data analyses: None 2. Modeling method: Underlying technology algorithm	Natural Cycles app 1. Data transmission: Anonymously enter data 2. Usage: Identify ovulation day and fertile window	Detect ovulation 1. Mean delay from ovulation day 2. Length of luteal phase 3. False-positive rate
Ecochard et al.^(20)^	2015	Canada	Observational study	107 women aged 19–45 years, included 326 cycles	1. BBT: Basal thermometer 2. Cervical mucus: Self-record sensation, appearance, and consistency 3. Hormonal assays: Estrone-3-glucuronide (E1-3-G), pregnanediol-3a-glucuronide (PDG), LH, and FSH levels 4. Reference standard for ovulation: Ultrasound investigations	Stress, illness, insomnia, disturbed sleep	1. Three-over-six rule 2. Data analyses and visualizations: R software 3. Modeling method: Last fertile sign algorithm	Diagram 1. Data transmission: Manual drawing 2. Usage: Identify fertile window	Fertile window Cervical mucus 1. Sensitivity 2. Specificity
Goodale et al.^(21)^	2019	Switzerland	Prospective observational Study	237 women mean 33 years, included 708 quilted cycles	1. Nocturnal wrist skin temperature: Ava bracelet, a wearable device 2. Physiological parameters (heart rate, HRV, respiratory rate, and skin perfusion): Ava bracelet 3. Sleep quality: Ava bracelet 4. Reference standard for ovulation: ClearBlue Digital Ovulation Test, a home-based urine LH test	In the 3 hours preceding sleep, had sexual intercourse, exercised heavily, eaten, drank coffee, or consumed alcohol	1. Data correction: Bonferroni method 2. Data processing and analysis: R software 3. Modeling method: Machine learning algorithm	Corresponding smartphone app 1. Data transmission: Synchronize data 2. Usage: Detect fertile window	Fertile window Accuracy
Scherwitzl et al.^(22)^	2016	Switzerland	Retrospective study	6,944 women mean aged 29 years, included 272,204 cycles	1. BBT: Basal thermometer 2. Reference standard for ovulation: LH tests 3. Date of menstruation	Sexual activity and personal notes	Modeling method: Underlying technology algorithm	Natural Cycles app 1. Data transmission: Anonymously enter data 2. Usage: Prevent pregnancy	1. Kaplan-Meier life table 2. Perfect-use Pearl Index 3. 13-cycle typical-use failure rate 4. 13-cycle typical-discontinuation rate 5. Discontinuation rate
Demian‘czyk and Michaluk^(23)^	2016	Poland	Retrospective study	361 women, included 17,322 cycles (age not stated)	1. BBT: Temperature sensor 2. Cervical mucus: Self-record the sensation 3. Date of menstruation	Contraceptive method Use of condoms, coitus interruptus, Creighton Model, contraceptive pill, thermometer, or contraceptive inserts and observation of mucus	1. Data processing and analysis: R software 2. Modeling method: Lady-Comp models	Lady-Comp, Pearly, and Daysy cycle computers 1. Data transmission: Anonymously enter data 2. Usage: Indicate fertile and infertile phases of menstrual cycle for contraception	2010 Pearl Index 2016 Pearl Index Percentage of planned pregnancies in women
Manhart and Duane^(24)^	2022	USA	Prospective cohort study	20 women aged < 40 years, included 240 cycles	1. BBT: Basal thermometer 2. Reference standard for ovulation: Peak mucus (2 days before and after)	None	1. Missing data: Missing data in a cycle were not entered 2. Modeling method: Natural Cycles algorithm	Natural Cycles app CycleProGo app 1. Data transmission: Anonymously enter data 2. Usage: Define fertile window	Equivalent 1. Fertile-window start and end day Natural Cycles app vs CycleProGo app 2. Mean overall fertile-window length 3. Cycles with a fertile-window start 4. Cycles with a fertile-window end
Fukaya et al.^(25)^	2017	Japan	Prospective cohort study	20 women, included 27 cycles (age not stated)	1. BBT: Conventional thermometer or wearable sensor 2. Date of menstruation Accuracy of the sequential prediction 1. Conventional calendar calculation method 2. Sequential predictive method	None	1. Conditional distributions of the phase: Sequential Bayesian filtering techniques 2. Missing data: Akaike information criterion (AIC) model 3. Modeling method: State-space model	Ran’s story website, a novel statistical framework 1. Data transmission: Entered data 2. Usage: Estimate menstrual cycle	Prediction accuracy State estimation and calculation of log-likelihood 1. Root mean square error (RMSE) 2. Mean absolute error (MAE)
Kawamori et al.^(10)^	2019	Japan	Retrospective study	3533 women aged 15–54 years, included 25,622 cycles	1. BBT: Conventional thermometer or a wearable sensor 2. Date of menstruation	Age Classified each menstrual cycle into eight age groups	1. Conditional distributions of the phase: Sequential Bayesian filtering techniques Identification of stages of the cycle 2. Modeling method: Self-excited threshold autoregressive state–space model	Ran’s story website 1. Data transmission: Enter data 2. Usage: Estimate menstrual cycle	State estimation and calculation of log-likelihood RMSE
Freundl et al.^(26)^	2014	Germany	Retrospective study	51 women aged 24–35 years, included 364 cycles	1. BBT: Conventional thermometer or wearable sensor 2. Date of menstruation	Mistakes or variations in the measurement method, measurements at different times, short or disturbed sleep, retiring late, unaccustomed amounts of alcohol, and emotional strain	1. Missing data: Missing data in a cycle were not entered 2. Modeling method: Tracking signal algorithm 3. Conventional method: Sensiplan® symptothermal method	Trigg’s tracking system (TtS) 1. Data transmission: Enter data 2. Usage: Define fertile window	TtS transition day and Sensiplan® initial day
van de Roemer et al.^(27)^	2021	Switzerland	Retrospective study	5328 women mean age 30 years, included 107,020 cycles	1. BBT: Basal thermometer 2. Date of menstruation	Age, BMI, cycle length, measurement skipping, high vs. low average temperature, and temperature steps	1. Missing data: Missing data in a cycle were not entered 2. Data processing and analysis: VE Analyzer 3. Modeling method: Fertility Tracker algorithm	Daysy, a fertility tracking device 1. Data transmission: Enter the data 2. Usage: Define the fertile window	Sensitivity analysis 1. Mean cycle length reported 2. Utilization rate 3. Fertility device identified on average
Stanford et al.^(28)^	2020	USA	Prospective cohort study	8363 women aged 21–45 years, included 200,712 cycles	1. BBT: Basal thermometer 2. Cervical fluid or cervix position: Self-record the sensation 3. Reference standard for ovulation: Urine LH test	Age, race/ethnicity, prior pregnancy, BMI, income, current smoking, education, partner education, caffeine intake, and use of hormonal contraceptives	1. Missing data: Use multiple imputation for imputing missing data	Mobile computing apps: Clue, Fertility Friend, Glow, Kindara, and Ovia-selected apps 1. Data transmission: Questionnaire survey 2. Usage: Track menstrual cycle and fertile window	1. Utilization rate 2. Fecundability ratios (FRs) (1) Increased fecundability 3. Time to pregnancy (TTP)
Shilaih et al.^(29)^	2018	Switzerland	Observational clinical study	136 women aged 20–40 years, included 437 cycles	1. Wrist-skin temperature (WST): Ava bracelet (wearable device) while sleeping 2. Reference standard for ovulation: ClearBlue Digital Ovulation Test, a home-based urine LH test	Consuming meals, drinking coffee, drinking alcohol, and engaging in sexual intercourse or heavy exercise	Principle: Three-over-six rule. 1. Data preprocessing: LOESS used to smooth temperature data 2. Data analyses and visualizations: R software 3. Modeling method: Linear mixed effects models	Ava app, a computerized fertility tracker 1. Data transmission: Synchronize data 2. Usage: Detect ovulation	1. Temperature shift rate 2. Temperature shift time 3. Temperature shift
Alzueta et al.^(9)^	2022	USA	Prospective observational study	26 women aged 18–35 years, included 416 cycles	1. Nocturnal distal skin temperature: Oura Ring, a multi-sensor wearable device 2. Physiological parameters (HR, HRV, and sleep): Oura Ring 3. Sleep quality: Subjective daily diary 4. Date of menstruation 5. Reference standard for ovulation: Urine LH test	Sleep quality, mood, readiness, and physical symptoms	1. Estimating menstrual cycle: Schmalenberge method 2. Modeling method: Hierarchical linear regression models	Oura apps 1. Data transmission: Anonymously synchronize data 2. Usage: Estimate menstrual cycle	Different phase: Menses, ovulation, mid-luteal, and late-luteal 1. Distal skin temperature 2. Correlation coefficient
Luo et al.^(30)^	2020	China	Prospective cohort study	34 women aged 22–42 years, included 125 cycles	1. Nocturnal ear inside temperature: Earpiece, an in-ear and non-invasive wearable device 2. BBT: Basal thermometer	Phlogistic illness and environmental situations	1. Data preprocessing: A data-cleaning protocol 2. Algorithm: An effective and flexible statistical learning algorithm 3. Modeling method: Hidden Markov Model (HMM)	Smartphone applications 1. Data transmission: Enter data 2. Usage: Detect ovulation	Earpiece vs. traditional method 1. Detection accuracy 2. Prediction power
Maijala et al.^(31)^	2019	Finland	Prospective cohort study	22 women aged 21–49 years, included 66 cycles	1. Nocturnal distal skin temperature: Oura Ring, a multi-sensor wearable device 2. BBT: Basal thermometer 3. Reference standard for ovulation: Urine LH test	Sleep quality	1. Data preprocessing: MATLAB script 2. Algorithm: HALF _ LOCS MENSES predict ovulation for middle day HALF PEAKS predict ovulation for first day	Oura apps 1. Data transmission: Synchronize data 2. Usage: Predict menstruation and ovulation cycles	1. Range of temperature increase in the luteal phase 2. Correlation between skin and oral temperatures 3. Sensitivity for menstruation 4. Length of fertile window
Gombert-Labedens et al.^(32)^	2024	USA	Prospective observational study	120 women aged 18–52 years, included 120 cycles	1. Nocturnal distal skin temperature: Oura Ring, a multi-sensor wearable device 2. Reference standard for ovulation: Urine LH test	Ambient temperature and humidity, basal metabolic rate, muscle activity, digestion, sleep, posture, and hormonal fluctuations	1. Data preprocessing: MATLAB script 2. Data analyses and visualizations: R software 3. Modeling method: Cosinor models	Oura apps 1. Data transmission: Anonymously synchronize data 2. Usage: Estimate menstrual cycle	Fit quality (r2) Menstrual cycle distal skin temperature data
Hurst et al.^(33)^	2022	USA	Prospective cohort study	80 women with ovulatory dysfunction aged 22–46 years, included 205 cycles	1. Overnight vaginal temperature: Vaginal sensor (VS) 2. Nocturnal WST: Skin-worn sensor 3. Reference standard for ovulation: Ultrasound ovarian follicle measurements and urine LH test	None	1. Principle: Three-over-six rule. 2. Algorithm: SWS and VS algorithm 3. Data processing and analysis: Days Difference Method and a threshold method	Smartphone applications 1. Data transmission: Anonymously synchronize data 2. Usage: Predict ovulation and conception	SWS vs VS 1. Accurate 2. Fertile window 3. Trying to conceive (TTC) time
Regidor et al.^(34)^	2018	Germany	Prospective cohort study	158 women aged 18–45 years, included 470 cycles	1. Circadian and circamensual core body temperature: Ovula Ring, a vaginal sensor 2. Hormonal assays: LH, follicle-stimulating hormone, estradiol, and progesterone levels 3. Vaginal ultrasound	None	1. Data expressed in form of a Cyclo Fertilo gram (CFG). 2. Mathematical algorithm based on circadian and circamensual core body temperature rhythm analysis	Smartphone applications 1. Data transmission: Synchronize data 2. Usage: Estimate menstrual cycle	1. Validation error due to software errors 2. Accuracy for detection of ovulation 3. Usage rate
Wark et al.^(35)^	2015	Australia	Comparative Observational Study	16 women aged 18–25 years, included 8 cycles	1. Upper arm temperature: BodyMedia SenseWear (BMSW) (Armband device) 2. BBT: Digital oral thermometer	None	1. Date visually and quantitatively: MTM 2. Data analyses and visualizations: Bland-Altman (BA) plot	WomanLog Pro app 1. Data transmission: Enter data 2. Usage: Monitor ovulation	1. Range of temperatures of thermometer 2. Acceptability of both devices

### Study Characteristics

The studies published from 2014 to 2024 were conducted in nine countries, including six in Switzerland; five in the United States, two each in China, Japan, and Germany; and one each in Canada, Poland, Finland, and Australia. The research participants were healthy childbearing women aged 18 and 45 years. Only one study focused on women with ovulatory dysfunction, who were studied to estimate the menstrual cycle and detect the fertile window and ovulation day. Regarding the research design, most studies were prospective cohort (n = 12, 57.1%) or retrospective (n = 6, 28.6%) studies, with the observation duration four to seven cycles per participant; five studies were large-scale studies, and three studies involved questionnaire surveys.

### Quality Assessment

Based on the 2024 CASP checklist assessment of the 21 included studies, this scoping review identified a tripartite stratification in methodological quality. Five studies (23.8%) raised major concerns, primarily due to incomplete confounding factor adjustments (Q5-Q6), as observed in Gombert-Labedens et al.^(32)^ and Hurst et al.^(33)^, which lacked clarity in addressing variables, as well as Manhart and Duane^(24)^ and Fukaya et al.^(25)^, which failed to account for these factors, coupled with issues such as unclear recruitment processes (Q2) in Hurst et al.^(33)^ or insufficient follow-up duration/completeness (Q7-Q8) in Manhart and Duane^(24)^. Three studies (14.3%) exhibited minor concerns, including those related to inconsistent outcome measurement methods (Q4) in Freundl et al.^(26)^, partial reporting of precision estimates (Q10) in van de Roemer et al.^(27)^, and uncertainties about local applicability (Q12) in Shilaih et al.^(29)^ and Freundl et al.^(26)^, with the latter and Regidor et al.^(34)^ additionally neglecting to conduct stratification/multivariate analyses for confounding controls. Thirteen studies (61.9%) demonstrated no concerns in adhering to robust standards, particularly in defining research questions (Q1: 100% compliance), measuring outcomes (Q4: 81% compliance), and presenting results (Q9–Q11: 76–100% compliance). However, pervasive gaps persisted, quantified in Table 3 as suboptimal confounding control compliance (Q5–Q6: 61.9%, with 8 studies deficient), follow-up completeness limitations (Q7–Q8: 71.4% compliance), and insufficient local applicability validation (Q12: 47.6% compliance), collectively underscoring the necessity for future research to prioritize prespecified confounding adjustments, extended follow-up ≥ 6 menstrual cycles, and diversified population sampling to enhance evidence reliability and clinical utility. The overall assessment of each study is presented in Table 3.

**Table 3:** CASP checklist summary of included studies.

	Zhu et al.^(8)^	Yu et al.^(18)^	Scherwitzl et al.^(19)^	Ecochard et al.^(20)^	Goodale et al.^(21)^	Scherwitzl et al.^(22)^	Demianczyk and Michaluk^(23)^
Q1	Yes	Yes	Yes	Yes	Yes	Yes	Yes
Q2	Yes	Yes	Can’t Tell	Yes	Yes	No	Can’t Tell
Q3	Yes	Yes	Yes	Yes	Yes	Can’t Tell	Yes
Q4	Yes	Yes	Yes	Yes	Yes	No	yes
Q5	Yes	Yes	No	No	Yes	No	No
Q6	No	Yes	No	No	Yes	No	No
Q7	Can’t Tell	Yes	No	No	Yes	No	Yes
Q8	Yes	Yes	No	Yes	Yes	No	Yes
Q9	Yes	Yes	Yes	Yes	Yes	Yes	Yes
Q10	Yes	Yes	Can’t Tell	Yes	Yes	Yes	Can’t Tell
Q11	Yes	Yes	Yes	Yes	Yes	Can’t Tell	Yes
Q12	No	Can’t Tell	Can’t Tell	Yes	Can’t Tell	No	Can’t Tell
Q13	Yes	Yes	Yes	Yes	Yes	Yes	Yes
Q14	Yes	Yes	Yes	Yes	Yes	Can’t Tell	Yes
	**Manhart and Duane** ^(24)^	**Fukaya et al.** ^(25)^	**Kawamor** ^(10)^	**Freundl et al.** ^(26)^	**van de Roemer et al.** ^(27)^	**Stanford et al.** ^(28)^	**Shilaih et al.** ^(29)^
Q1	Yes	Yes	Yes	Yes	Yes	Yes	Yes
Q2	Yes	Yes	Can’t Tell	Yes	Yes	Yes	Yes
Q3	Can’t Tell	Can’t Tell	Yes	Yes	Yes	Can’t Tell	Yes
Q4	Yes	Yes	Yes	Can’t Tell	Yes	Yes	Yes
Q5	No	No	No	No	No	Yes	Yes
Q6	No	No	Can’t Tell	No	No	Yes	Yes
Q7	Yes	Yes	Can’t Tell	Yes	Yes	Yes	Can’t Tell
Q8	Yes	Yes	Yes	Yes	Yes	Yes	Yes
Q9	Yes	Yes	Yes	Yes	Yes	Yes	Yes
Q10	Yes	Yes	Yes	Yes	Yes	Yes	Yes
Q11	Yes	Yes	Yes	Yes	Yes	Yes	Yes
Q12	Can’t Tell	No	Can’t Tell	Can’t Tell	Can’t Tell	No	Yes
Q13	Yes	Can’t Tell	Yes	Yes	Yes	Yes	Yes
Q14	Yes	Yes	Yes	Yes	Yes	Yes	Yes
	**Alzueta et al.** ^(9)^	**Luo et al.** ^(30)^	**Maijala et al.** ^(31)^	**Gombert-Labedens et al.** ^(32)^	**Hurst et al.** ^(33)^	**Regidor et al.** ^(34)^	**Wark et al.** ^(35)^
Q1	Yes	Yes	Yes	Yes	Yes	Yes	Yes
Q2	Yes	Can’t Tell	Can’t Tell	Yes	No	Can’t Tell	Can’t Tell
Q3	Yes	Yes	Yes	Yes	Yes	Yes	Yes
Q4	Yes	Can’t Tell	Yes	Can’t Tell	Yes	Yes	Yes
Q5	No	No	Yes	No	Yes	No	No
Q6	Can’t Tell	Yes	Can’t Tell	No	Yes	No	No
Q7	Yes	Yes	Yes	Yes	No	Yes	Yes
Q8	Yes	Yes	Yes	No	Yes	Yes	Yes
Q9	Yes	Yes	Yes	Yes	Yes	Yes	Yes
Q10	Yes	Yes	Yes	Can’t Tell	Yes	Can’t Tell	Can’t Tell
Q11	Yes	Can’t Tell	Yes	Yes	Yes	Yes	Yes
Q12	Yes	Can’t Tell	Can’t Tell	Can’t Tell	Can’t Tell	Can’t Tell	Can’t Tell
Q13	Yes	Yes	Yes	Yes	Yes	Yes	Yes
Q14	Yes	Yes	Yes	Yes	Yes	Yes	Yes

1. Did the study address a clearly focused issue?

2. Was the cohort recruited in an acceptable way?

3. Was the exposure accurately measured to minimize bias?

4. Was the outcome accurately measured to minimize bias?

5. Have the authors identified all important confounding factors?

6. Have they taken account of the confounding factors in the design and/or analysis?

7. Was the follow up of subjects complete enough?

8. Was the follow up of subjects long enough?

9. What are the results of this study?

10. How precise are the results?

11. Do you believe the results?

12. Can the results be applied to the local population?

13. Do the results of this study fit with other available evidence?

14. What are the implications of this study for practice?

### Measurement Content and Methods

Nine studies analyzed the use of wearable devices, including bracelet, ring, vaginal, ear, and armband devices, for measuring body temperature (Table 4). These novel wearable devices continuously and automatically measure skin temperature during sleep to define BBT, with data collected from the middle phase of the night to avoid disturbances from the falling asleep and waking up phases^(8)^. Furthermore, 16 studies considered some confounding factors that influence BBT, including individual factors (e.g., age, weight, illness, and medications), psychosocial factors (e.g., stress, insomnia, and climate change), and lifestyle factors (e.g., sexual activity, smoking status, and alcohol and coffee consumption). Furthermore, Yu et al.^(18)^ and Alzueta et al.^(9)^ used multi-sensor wearable devices (the Huawei Band and Oura Ring) to measure physiological parameters (heart rate [HR], HR variability [HRV], and sleep) to enhance prediction accuracy. Other studies used a basal thermometer to discuss how to improve the prediction accuracy by updating the algorithm.

**Table 4:** Wearable device characteristics and functions.

Type of device	Bracelet device	Ring device	Vaginal device	Ear device	Armband device
Product Name	Ava bracelet	Oura Ring	Ovula	Earpiece	BodyMedia SenseWear
Measurement	Wrist skin temperature (WST)	Distal skin temperature (DST)	Core body temperature (CBT)	Ear inside temperature (EIT)	Upper armband temperature (UAT)
Characteristics	1. Recording frequency: Every 10 s throughout night 2. At least 4 hours of uninterrupted sleep	1. Recording frequency: Every 30 s throughout night 2. Every 30 s of sleep monitoring throughout night	1. Recording frequency: Every 5 min throughout entire cycle 2. Circadian and circamensual intravaginal exact measurement	1. Recording frequency: Every 5 min throughout night 2. At least delete initial 20–30 min of data to stabilize	1. Recording frequency: Every 5 min throughout night 2. Delete 60 min of data before waking
Research Content	1. Prediction of the fertile window 2. Tracking the menstrual cycle	Tracking the menstrual cycle	Prediction of fertile window and ovulation	Prediction of ovulation	Prediction of ovulation

### User Experience of Wearable Devices

In the current technological landscape of fertility monitoring wearable devices, five categories (bracelets, rings, vaginal sensors, ear-worn devices, and armbands) demonstrate distinct performance variations and user-specific adaptability. Bracelets enable unobtrusive continuous tracking through wrist skin temperature monitoring with strong resistance to environmental interference, yet require daily charging and pose potential wrist movement restrictions. Nevertheless, they are suitable for women planning pregnancy who prioritize convenience, although sleep-related discomfort may compromise compliance^(8,21,29)^. Featuring waterproof capabilities (up to 50 meters water resistant and sauna compatible), high data integrity (> 97%), and 3-day battery life, rings cater to women with irregular schedules, but strict size constraints and manual synchronization dependencies risk operational oversights^(9,31,32)^. Vaginal sensors provide precise core body temperature measurements without urine sampling, ideal for cross-time zone or shift workers, yet their invasive design, which is associated with menstrual discomfort and psychological barriers, and requirement for monthly replacement limit their usage primarily to patients with infertility requiring high-precision data^(34)^. Ear-worn devices attract tech-savvy users through stable ear canal temperature monitoring and fully automated artificial intelligence analysis, although their tendency to detach during sleep and risk of ear canal irritation may lead to nocturnal data gaps^(18,30)^. With their 7-day battery life, minimal skin irritation, and multi-parameter monitoring, armbands serve clinical trial contexts but face practical limitations due to a 35% missed measurement rate and reliance on expert data interpretation^(35)^.

Overall, device selection necessitates balancing accuracy (optimal in vaginal sensors) and compliance (better in bracelets and rings), alongside considerations of psychological acceptability (higher for non-invasive devices) and scenario-specific demands (medical-grade monitoring often sacrifices convenience). Future research should prioritize technical optimizations (e.g., extended battery life and reduced invasiveness) and personalized adaptations (based on occupational or physiological tolerance) to enhance long-term reliability and user adherence. These considerations for wearable devices are presented in Table 5.

**Table 5:** User experience considerations for wearable devices.

Device type	Advantages	Disadvantages	Target users	Negative experiences
Bracelet	1. Unobtrusive continuous monitoring (wrist skin temperature) 2. Strong resistance to environmental interference 3. Automatic data sync with app	1. Daily charging required 2. Potential wrist movement restrictions	Women seeking convenient fertility tracking	Discomfort while wearing during sleep
Ring	1. Waterproof (50 m depth) and sauna compatible 2. > 97% data availability 3. 3-day battery life	1. Daily manual data sync required 2. Strict finger size requirements	Women with jobs with irregular schedules	Risk of forgetting synchronization routines
Vaginal	1. Most accurate core temperature measurement 2. No urine sampling needed 3. Adaptable to shift workers/time zone travelers	1. Potential menstrual discomfort 2. Monthly replacement required 3. Psychological resistance	Infertility patients needing precision	Psychological barriers to insertion
Ear	1. High ear canal temperature stability 2. Fully automated monitoring 3. AI-powered app analysis	1. Easy detachment during sleep 2. Irritation risk for sensitive users	Early tech adopters	Data gaps from nighttime movement
Armband	1. Long battery life (7 days) 2. Low skin irritation 3. Multi-parameter monitoring	1. Low compliance (35% missed measurements) 2. Requires expert data interpretation	Clinical trial participants	Restricted arm movement perception

### Long-Term Monitoring Analysis and Clinical Implications

The existing literature indicates that temperature monitoring using wearable technology presents considerable advantages and research opportunities for the management of menstrual cycles over extended periods. The Ava bracelet, in particular, demonstrated significant fluctuations in wrist skin temperature (WST), heart rate, and respiratory rate across a year of continuous monitoring. This investigation confirmed that machine learning algorithms can achieve an accuracy rate of 90% in predicting the fertile window by synthesizing multi-parametric data, thereby highlighting the importance of long-term data for the optimization of these algorithms^(21)^.

The study examining the Daysy device emphasized the critical importance of user compliance; specifically, when the frequency of measurements surpassed 80%, the accuracy of the algorithm’s outputs improved significantly, with 42.4% of “green days” accurately identified within fertile windows. Furthermore, the stability of the luteal phase length was maintained (mean of 12.7 ± 1.4 days), underscoring the necessity of consistent monitoring for the effectiveness of natural contraception methods^(27)^. WST monitoring further validated its potential as an alternative to oral BBT measurement by exhibiting a biphasic pattern that correlated with oral BBT (r = 0.563) and a 0.30°C elevation during the luteal phase compared with follicular phase temperatures, although challenges related to environmental interference persisted^(29)^. Additionally, the application of cosine modeling to long-term BBT data successfully identified 82% of biphasic cycles by quantifying rhythmic features such as median, amplitude, and peak phase, while also flagging abnormal cycles through deviations in these parameters, thus providing a quantitative tool for assessing menstrual health^(32)^. In summary, long-term monitoring not only clarifies the dynamic stability of physiological parameters, such as the consistency of luteal phase temperature and length, but also reveals technical limitations, including device variability and environmental confounders.

### Complex Data Processing and Analysis

Nine studies collected data simultaneously via Bluetooth synchronization, while the others relied on manual data entry by the participants, which may have led to input errors. To protect patient privacy and masking of subgroup differences, the data were collected in an anonymous form in seven studies. Due to the dense and complex nature of the temperature data, some studies conducted data preprocessing. For example, according to a data-cleaning protocol, the data were cleaned and organized to ensure accuracy, consistency, and completeness^(30)^, and a MATLAB script was developed to manage it^(31)^. In addition, each parameter underwent locally estimated scatterplot smoothing (LOESS) before statistical analysis^(19)^. Regarding missing data, five studies excluded it, and one study used multiple imputations for imputing the missing data^(28)^. In terms of data analysis, some research presented data in the form of graphs and charts to better analyze it^(34,35)^. Four studies explained how BBT predicted ovulation based on the “three over six” rule, which means that three temperatures are required to be 0.2°F above the highest point of the previous six temperatures, with at least one of the higher temperatures being 0.4°F above the lower ones^(2)^. Freundl et al.^(26)^ used Sensiplan® symptom-thermal methods to further enrich the rule. Additionally, 33.3% of studies used R software for data analyses and visualizations, 23.8% constructed linear mixed effects models, and 76.2% adopted innovative algorithms and models for prediction. Further exploration is needed to improve the accuracy of the predictions.

### Functionality and Effectiveness of the Applications

The main functions of the applications include four aspects: menstrual cycle estimation (8 applications), fertility prediction (14 applications), contraception indication (2 applications), and pregnancy monitoring (1 application). Currently available applications include Ava, Natural Cycles, and Oura, WomanLog Pro, as well as the Ran’s Story website, and Pearly and Daysy Cycle computers. Stanford et al.^(28)^ evaluated the fecundability of five applications (Clue, Fertility Friend, Glow, Kindara, and Ovia) and reported them as effective, but the findings lacked evidence. The evaluation metrics for application effectiveness were temperature shift; fertility window length; and model performance, including accuracy, sensitivity, specificity, root mean square error (RMSE) mean absolute error (MAE), correlation coefficient, fertility index (Pearl Index, fecundability ratios, and time to pregnancy), and app availability (utilization and discontinuation rates).

## DISCUSSION

The majority of studies examined in this review demonstrated that BBT can predict ovulation, and BBT is recognized as an indicator of fertility^(36)^. However, traditional BBT measurement requires fixed timing and location, typically every morning before arising, which involves manually entering data into an application and measuring body temperature to track menstruation and ovulation^(37,38)^. This daily routine can be tedious and subject to reporting errors^(36,39)^. In this scoping review, 42% of the research was found to focus on using wearable devices to predict menstrual cycles and ovulation, driven by advancements in portable sensors and wearable technology that allow for continuous and dynamic collection of health information throughout the day^(40,41)^. The results of this study confirm that wearable devices that continuously measure skin temperature and automatically synchronize data appear to be highly suitable for addressing the current limitations of traditional and BBT-based tracking apps^(22)^. These devices can be worn on various parts of the body—the wrist, finger, upper arm, inside the vagina, or in the ear—and enable more extensive longitudinal tracking of physiological parameters, allowing users to observe personalized patterns in the evolving data^(22,42)^. Moreover, these devices can also assess overall physical condition by measuring physiological changes, such as changes in HR, HRV, respiratory rate, skin perfusion, and sleep quality^(9,19,22)^. Overall, compared to conventional devices, wearable devices have the potential to enhance BBT tracking by continuously measuring multiple physiological parameters, leading to a more precise estimation of ovulation. Further research is needed to validate these advancements. The main conclusions and clinical implications by device type are listed in Table 6.

**Table 6:** Clinical significance of different device types.

Device type	Clinical significance	Key conclusions
Bracelet	1. Continuous, unobtrusive tracking of ovulatory BBT trends 2. Anti-interference design enhances reliability of basic fertility assessment	**Pros:** Universal applicability for daily fertility management prioritizes convenience **Cons:** Sleep discomfort causes 23% attrition **Indication:** Primary screening tool for general trying to conceive populations
Ring	1. Waterproof design enables data capture in special environments (e.g., when swimming, in sauna) 2. High data integrity improves cycle prediction accuracy	**Pros:** Extended battery and waterproof features suit high-stress occupational groups **Cons:** Size limitations exclude 5–15% of women (finger circumference mismatch) **Indication:** Long-term monitoring for populations with irregular schedules
Vaginal	1. Core body temperature precision (±0.05°C, gold-standard level) 2. Eliminates morning urine sampling errors	**Pros:** Provides medical-grade data for Assisted Reproductive Technology interventions **Cons:** > 40% attrition due to psychological barriers, especially for first-time users **Indication:** Essential for precision monitoring in ART cycles
Ear	1. Ear canal temperature stability exceeds skin surface by 0.1–0.3°C AI algorithms increase abnormal ovulation detection rates	**Pros:** Automated analysis reduces user cognitive burden **Cons:** Nighttime dislodgement causes critical data loss (~30%) **Indication:** Personalized fertility management for tech-engaged users
Armband	1. Multiparameter analysis (temperature + heart rate variability) 2. Extended battery supports chronic fertility disorder research	**Pros:** Superior skin biocompatibility (< 2% hypersensitivity) **Cons:** High data loss rates limit clinical utility **Indication:** Physiological mechanism exploration in research settings

The fusion of wearable sensor technology with machine learning algorithms has significantly advanced the development of intelligent fertility tracking applications^(43)^. This integration has the potential to enable patient comprehensive characterization and optimized clinical interventions^(44)^. Critical to realizing this vision is an accurate estimation of BBT time-series data, and several scholars have tried to remedy any deficiencies. For example, Fukaya et al.^(25)^ and Kawamori et al.^(10)^ developed a state space model that includes the menstrual phase as a latent state variable to explain daily fluctuations in BBT and the menstruation cycle length. The state space model relies on sequential Bayesian filtering techniques to map data to a state space to capture long-term dependency relationships and is commonly employed for describing and analyzing the behavior of dynamic systems in academic research^(45)^. Similarly, Luo et al.^(30)^ used a hidden Markov model to describe the probabilistic relationship between observation and hidden state sequences to predict future observation results or classify a sequence according to the potential hidden process of generated data. To further analyze the skin temperature circadian rhythm, some studies applied the cosinor model to facilitate the evaluation of menstrual cycle effects on physiological parameters and in clinical settings, using the characteristics of menstrual cycles as health markers or to facilitate menstrual chronotherapy^(46)^. Some presented data analysis results in a visual format, helping users better understand the findings^(22)^. The underlying technology algorithm was based on data entered each day, and the results were presented as either a red (risk) or green (no risk) icon to indicate risk of pregnancy^(22)^. Machine learning algorithms will continue to offer more possibilities for improving the accuracy of fertility tracking applications in the future.

A web-based pilot survey found that approximately a quarter of respondents reported using fertility tracking applications, and 63% of users agreed that the applications were science-based and successful in determining the fertile window^(47)^. However, the sensitivity of fertility tracking applications varies widely, from 62% for wrist-worn sensors to 28% for ear-worn devices^(30,48)^. Moglia et al.^(49)^ used the APPLICATIONS scoring system to assess menstrual cycle tracking applications and found that most free smartphone applications were inaccurate^(50)^. Many fertility-related apps may be less accurate and less likely to publish their data in peer-reviewed journals. Therefore, reliance on these applications may lead to unintended pregnancies or delay in identifying infertility issues, causing indelible consequences^(7,39,47)^. Further exploration of the user experience also revealed that users had concerns regarding inaccurate forecast dates, cost, and data privacy, as well as anxiety and frustration over unreliable prediction of the menstrual cycle^(8,51)^. As application usage was observed to gradually decrease over time, the means of enhancing adherence while improving prediction accuracy are issues worth studying.

Women using these applications expect to receive scientific and comprehensive reproductive health information as well as emotional support and advice on contraceptive decisions, sexual health, and postoperative care^(47)^. However, one review found that not all available applications are evidence-based^(37)^, and only 17% provide information on contraception^(49)^. Therefore, application designers should consider the diverse and evolving needs of users, catering to a wider range of purposes such as contraception and fertility tracking while observing the response to medication, monitoring reproductive diseases, and reaching out to a more diverse audience^(5,7)^. To obtain more accurate fertility information, it is crucial to provide preliminary training on measurement methods and application operating steps. For example, personalized symptothermal method training can be obtained through the International Couple to Couple League (https://ccli.org/)^(52)^.

## STUDY LIMITATIONS

The current body of evidence on BBT-based fertility tracking technology, while promising, exhibits significant heterogeneity and methodological limitations that necessitate cautious interpretation. As identified through the CASP quality assessment, key concerns impacting the rigor and comparability of findings include the fact that a substantial proportion of studies inadequately addressed or reported on confounding factors known to influence BBT (e.g., illness, medication, significant lifestyle changes, and sleep disturbances). This omission introduces bias and limits the ability to isolate the true effects of the tracking technology itself on outcomes such as ovulation prediction accuracy and contraceptive efficacy. Another limitation was that the study included diverse populations from different countries (e.g., Switzerland, the United States, China, and Japan) of varying age ranges who were both healthy and who had health conditions (e.g., ovulatory dysfunction). Although this reflects real-world diversity, it also contributes to heterogeneity in results. Baseline fertility status, cycle regularity, cultural factors influencing compliance, and access to technology varied considerably. A third limitation was that the studies utilized different wearable devices (wristbands, rings, vaginal sensors, and earpieces) and traditional thermometers, each with inherent measurement variability. Crucially, the reference standards for confirming ovulation also differed (urine LH tests, ultrasound, serum hormone level, and cervical mucus assessment), each with varying sensitivity and specificity. This heterogeneity makes direct comparison of application and device performance across studies challenging. Finally, although use of prospective cohorts was common, several key studies relied on retrospective designs or lacked sufficient follow-up duration or completeness, raising concerns about selection bias and attrition bias, with only five studies conducted on a large scale. These limitations highlight the need for high-quality, prospective studies utilizing standardized protocols to control confounding factors while employing robust reference standards (such as serial ultrasound and serum progesterone measurement), clearly reporting algorithm methodologies (or making them open-source), and including diverse populations representative of intended users.

## CONCLUSION

This review revealed several areas that require further research and upon which recommendations can be provided. First, wearable devices should collect data in a manner that avoids confounding the data with other factors and further improves the accuracy of the data. Second, innovative machine learning algorithms should process large sample time-series data and explore the best algorithm. Third, personalized, comprehensive, and scientific fertility information should be provided to women trying to conceive or prevent pregnancy. Fourth, focus should be placed on the user experience, interaction, and privacy. Fifth, the training and evaluation system of fertility tracking applications should be improved. Conducting further research is needed to apply these recommendations and enhance the value of fertility tracking based on BBT while promoting its further development.

## Data Availability

The entire dataset supporting the results of this study was published in the article and in the “Supplementary Materials” section.
