# Supplementary material for: Effectiveness of menstruation and fertility tracking technology in childbearing women: a scoping review
Source: Rev Esc Enferm USP. 2025 Oct 13;59:e20240454. doi: 10.1590/1980-220X-REEUSP-2024-0454en (PMC12519949; doi:10.1590/1980-220X-REEUSP-2024-0454en)
Supplement: Translation

# Translation: Eficacia de la tecnología de seguimiento de la menstruación y la fertilidad en mujeres en edad fértil: una revisión de alcance

**DOI:** 10.1590/1980-220X-REEUSP-2024-0454es

**Authors:** Yi Li, Yuhui Fang, Shuiqin Gu

**Affiliations:** 1 Zhejiang Chinese Medical University, Master Degree Cultivation Base, Zhejiang Province, China.; 2 Jiaxing University, Jiaxing Maternity and Children Health Care Hospital, Nursing Department, Jiaxing, Zhejiang Province, China.

**Keywords:** Detección de la Ovulación, Ventana Fértil, Menstruación, Embarazo, Revisión de Alcance

## Abstract

**Objetivo:**

Investigar protocolos estandarizados de medición de temperatura e inteligentes métodos de procesamiento de datos para mejorar la precisión de la predicción de la ovulación.

**Método:**

Basándose en el marco de informe de revisión de alcance de Arksey y O’Malley, se recuperaron publicaciones relevantes desde el 15 de agosto de 2014 hasta el 15 de agosto de 2024 de las bases de datos MEDLINE, EMBASE, SCOPUS y Web of Science. Las publicaciones se seleccionaron, resumieron y evaluaron según el Programa de Habilidades de Evaluación Crítica (CASP) para evaluar su rigor metodológico.

**Resultados:**

Se incluyeron un total de 21 publicaciones que informaban sobre estudios realizados en 9 países y que involucraron a 26.044 participantes. Los dispositivos de medición de sistemas de seguimiento de la fertilidad basados en la temperatura corporal basal (TBC) incluyeron dispositivos portátiles y termómetros de temperatura corporal basal. Las funciones de las aplicaciones abarcaron evaluación menstrual, predicción de fertilidad, anticoncepción y gestión del embarazo. Las aplicaciones fueron evaluadas en términos de funcionalidad y experiencia de usuario.

**Conclusión:**

La investigación sobre la aplicación del seguimiento de la fertilidad basado en la TBC se encuentra aún en etapa preliminar. Los hallazgos de este estudio brindan una valiosa referencia para el desarrollo de aplicaciones personalizadas y convenientes, lo cual requiere futuras investigaciones prospectivas de cohorte de alta calidad.

## INTRODUCCIÓN

La temperatura corporal basal (TCB) puede utilizarse como indicador de fertilidad y se utiliza comúnmente para estimar la ventana de fertilidad mediante un monitoreo constante, incluido el seguimiento digital en aplicaciones de salud (apps)^(1)^. El principio en el que se basa el monitoreo es que la TCB presenta un patrón bifásico debido al efecto termogénico de la progesterona, una hormona que aumenta después de la ovulación^(2)^. Actualmente, aproximadamente el 68% de las aplicaciones de seguimiento de la fertilidad se basan en datos auto-informados sobre la TCB y el ciclo menstrual para predecir el seguimiento menstrual y la fertilidad, ya que es un método rentable, fácil de usar y no invasivo^(3,4)^. Estas aplicaciones suelen incluir diversas funciones, como el seguimiento de los ciclos menstruales, el registro de síntomas, la anticoncepción y la planificación familiar, y el control del embarazo^(5)^. Sin embargo, a pesar de su popularidad, estas aplicaciones han sido criticadas por su potencial daño debido a la preocupación por su precisión y la falta de pruebas sólidas que respalden su eficacia, lo que puede dar lugar a riesgos potenciales como embarazos no deseados o retrasos en el diagnóstico de la infertilidad^(6,7)^. Por lo tanto, existe una necesidad urgente de mejorar la precisión predictiva de estas aplicaciones y reforzar las pruebas pertinentes.

Con el desarrollo de los dispositivos portátiles y los avances en los algoritmos de aprendizaje automático, la predicción precisa de la ventana de fertilidad es cada vez más factible. Cuando se llevan por la noche en diversas partes del cuerpo, como la muñeca o las zonas distales, los dispositivos portátiles pueden proporcionar datos continuos y detallados sobre la temperatura de la piel^(8,9)^. Múltiples estudios han confirmado la coherencia entre la temperatura cutánea nocturna y la TCB^(8,10)^. Al mismo tiempo, se han desarrollado innovadores algoritmos de aprendizaje automático para analizar amplios datos de series temporales con el fin de mejorar la precisión de la predicción del día de la ovulación^(8,11)^.

A pesar de estos avances, ninguna revisión sistemática ha evaluado el estado actual de los dispositivos de medición, la función de los algoritmos inteligentes o la eficacia de las aplicaciones de seguimiento de la fertilidad basadas en la TCB. Para abordar esta laguna, se propuso una revisión de alcance que pudiera identificar el progreso de la investigación en áreas temáticas específicas más rápidamente que las revisiones tradicionales, al tiempo que proporcionara una visión general que pudiera impulsar la actualización de los sistemas de conocimiento en este campo^(12)^. Basándose en esta propuesta, el presente estudio tuvo como objetivo realizar una revisión de alcance sistemática basada en el marco de revisión de alcance de Arksey y O’Malley para evaluar la precisión, las funcionalidades y la experiencia del usuario de las aplicaciones de seguimiento de la fertilidad que utilizan algoritmos inteligentes y se basan en la TCB. Los resultados proporcionan una referencia y una base para futuras investigaciones sobre las aplicaciones de seguimiento de la fertilidad basadas en la TCB entre mujeres en edad fértil.

## MÉTODO

### Diseño del Estudio

Esta revisión de alcance siguió el marco metodológico del Instituto Joanna Briggs (JBI), cuyo objetivo es proporcionar respuestas a una pregunta de investigación bien definida. Este marco, que incluye investigaciones de diversos diseños, describe el alcance, el ámbito y la naturaleza de la investigación e identifica las lagunas en la bibliografía existente. Consta de cinco pasos: alcance, búsqueda, selección, extracción de datos y análisis de datos^(13)^. La presentación de los métodos y los resultados se ha guiado por los criterios de los Elementos de información preferidos para las revisiones sistemáticas y los metaanálisis (PRISMA)^(14)^. Los datos para esta revisión eran de dominio público, por lo que no fue necesaria la aprobación del comité de ética de la institución.

### Preguntas de Investigación

Esta revisión de alcance aborda las siguientes preguntas de investigación: (1) ¿Cuáles son los dispositivos de monitoreo de la TCB y cómo se pueden controlar los factores de confusión? (2) ¿Cuál es el contenido específico de las aplicaciones de seguimiento menstrual y de la fertilidad? (3) ¿Cuáles son los efectos de la aplicación de aplicaciones de seguimiento menstrual y de la fertilidad?

### Estrategia de Investigación

La elegibilidad de los estudios se evaluó basándose en el marco de población, concepto y contexto (PCC) sugerido por el JBI^(15,16)^. La Tabla 1 ofrece una visión general de los criterios PCC y los tipos de evidencia considerados en esta revisión de alcance. Se realizaron búsquedas en las bases de datos MEDLINE, EMBASE, SCOPUS y Web of Science para encontrar todos los artículos en inglés publicados entre el 15 de agosto de 2014 y el 15 de agosto de 2024. La estrategia detallada en PubMed consistió en utilizar los siguientes encabezados de temas de salud (MESH) ((women AND (humans[Filter]) AND (“Basal Body Temperature”[Title/Abstract] OR “BBT”[Title/Abstract] OR “Skin Temperature”[Title/Abstract] OR “WST”[Title/Abstract] OR “core body temperature”[Title/Abstract] OR “thermal method”[Title/Abstract] OR “Symptothermal method”[Title/Abstract] OR “temperature Sensor”[Title/Abstract]) AND (“system*”[Title/Abstract] OR “device”[Title/Abstract] OR “app*”[Title/Abstract] OR “computers”[MeSH])) AND (2014:2024[pdat])). También se utilizó otra estrategia de búsqueda en bases de datos ideada por los investigadores y que cumplía con la lista de verificación de la Revisión por pares de estrategias de búsqueda electrónica (archivo adicional 1).

**Tabla 1:** Criterios de inclusión y exclusión basados en el marco de población, concepto y contexto.

	Criterios de inclusión	Criterios de exclusión
Población	Mujeres en edad fértil	Mujeres embarazadas o en periodo de lactancia
Concepto	Aplicaciones de seguimiento para predecir la menstruación y el periodo fértil	Centrarse únicamente en la experiencia del usuario y la usabilidad de la aplicación
Contexto	Diferentes aplicaciones de seguimiento de la menstruación y la fertilidad basadas en la temperatura corporal basal	No aplicable
Tipos de evidencia	ECA; cuasi-ECA; y estudios cualitativos, cuantitativos y de métodos mixtos	Revisiones, metaanálisis, experimentos con animales y artículos sin texto completo

ECA: ensayos controlados aleatorios.

### Extracción de Datos

Al mismo tiempo que se realizaba la selección, se elaboró una tabla de extracción de datos para recolectar información sobre los autores de cada estudio, el año de publicación, el país, el diseño de la investigación, el objetivo de la investigación, el método de medición de la TCB, los factores de confusión, el procesamiento y modelado de los datos, y el uso y la eficacia de la aplicación. Dos investigadores verificaron de forma independiente la exactitud de los datos de la tabla.

### Selección de Estudios

Tres investigadores establecieron un entendimiento común sobre el contenido y la eficacia de las aplicaciones de seguimiento menstrual y de la fertilidad. Dos investigadores seleccionaron de forma independiente los títulos y resúmenes de 1357 registros y compararon y debatieron los resultados de los artículos seleccionados que cumplían los criterios de inclusión, que se consideraron aptos para la selección del texto completo. Los investigadores solicitaron el asesoramiento de una tercera persona en caso de desacuerdo.

### Evaluación de la Calidad

Se realizó un análisis de calidad de la bibliografía incluida en la revisión de alcance para evaluar sus fortalezas y limitaciones. Se aplicó la herramienta Programa de habilidades de evaluación crítica 2024 (2024 Critical Appraisal Skills Programme-CASP) para facilitar una evaluación sistemática de los componentes metodológicos clave, incluyendo el diseño del estudio, la validez, el rigor y la relevancia para la pregunta de investigación^(17)^. Dos investigadores realizaron de forma independiente la evaluación de la calidad y posteriormente llegaron a un consenso sobre la calidad metodológica de los estudios.

La evaluación clasificó los estudios en tres categorías: sin preocupaciones (respuesta «Sí» a todos los criterios), preocupaciones menores (respuesta «Sí» a todos los criterios excepto uno, al que se respondió «No» o «No se decir») y preocupaciones importantes (respuesta «No» o «No se decir» a más de un criterio). Cualquier desacuerdo se resolvió mediante debate.

### Análisis de Datos

La síntesis y el análisis de los datos fueron realizados por dos revisores después de llegar a un consenso mediante debate. Las categorías iniciales se desarrollaron mediante un acuerdo final, y los datos extraídos se examinaron, debatieron, compararon y recopilaron. Las categorías se perfeccionaron y ajustaron mediante debate y reevaluación de los datos extraídos, y las categorías finales se nombraron y presentaron en formato tabular.

## RESULTADOS

### Resultados de la Búsqueda

Se identificaron inicialmente un total de 1547 estudios, de los cuales se eliminaron 190 por duplicados. La revisión de los títulos y resúmenes excluyó 1294 artículos, lo que dejó 63 artículos completos para su selección. De estos, se excluyeron 42 artículos: 13 porque eran artículos de revisión, 11 porque no eran coherentes con la definición de la BBT y 18 porque no informaban sobre aplicaciones de seguimiento, lo que dejó un total de 21 estudios elegibles para su revisión (Figura 1). Los detalles de estos estudios se muestran en la Tabla 2.

**Figura 1:** Diagrama de flujo de selección del estudio PRISMA.

**Tabla 2:** Datos extraídos de los 21 estudios incluidos.

Autor	Año de publicación	País	Diseño de la investigación	Participantes en la investigación	Contenido y método de medición	Variables de confusión	Procesamiento de los datos del estudio	Funcionalidad de la aplicación	Eficacia de la aplicación resultados
Zhu et al.^(8)^	2021	Suiza	Estudio prospectivo de cohortes	57 mujeres sanas de entre 18 y 45 años, se incluyeron 193 ciclos	1. Temperatura nocturna de la piel de la muñeca: pulsera Ava Fertility Tracker, un dispositivo wearable 2. TCB: Lady-Comp, un termómetro digital 3. Estándar de referencia para la ovulación: ClearBlue Digital Ovulation Test, una prueba de LH en orina para uso doméstico)	Edad, peso, altura, raza y tiempo desde que dejaron de tomar anticonceptivos hormonales	1. Procesamiento de datos: regla de tres sobre seis 2. Método de modelización: modelo lineal de efectos mixtos	Aplicación Ava, un registro de fertilidad informatizado 1. Transmisión de datos: sincronización anónima de datos 2. Uso: Detectar la ovulación	Temperatura de la piel de la muñeca frente a la TCB Detección de la ovulación 1. Sensibilidad 2. Especificidad 3. Probabilidad de que se haya detectado la ovulación 4. Valores predictivos negativos 5. Coeficiente de correlación de medidas repetidas 6. Rango de aumento de la temperatura
Yu et al.^(18)^	2022	China	Estudio prospectivo de cohortes	114 mujeres no embarazadas de entre 18 y 45 años, incluidos 382 ciclos	1. Frecuencia cardíaca: Huawei Band 5, un dispositivo wearable 2. TCB: Braun IRT6520, un termómetro de oído 3. Estándar de referencia para la ovulación: ecografía ovárica y niveles hormonales séricos	Edad, peso, altura, estado civil, nivel educativo, ocupación, edad en la menarquia, tabaquismo y consumo de alcohol	1. Corrección de datos: método Bonferroni 2. Análisis y visualización de datos: software R 3. Método de modelización: modelo lineal mixto	Aplicación Huawei 1. Transmisión de datos: sincronización de datos 2. Uso: Detectar el periodo fértil y la menstruación	Menstruaciones regulares frente a irregulares Ventana fértil y menstruación Precisión Sensibilidad Especificidad Área bajo la curva
Scherwitzl et al.^(19)^	2015	Suiza	Estudio retrospectivo	317 mujeres de entre 18 y 39 años, incluidos 1501 ciclos	1. TCB: termómetro basal 2. Estándar de referencia para la ovulación: pruebas de LH 3. Fecha de la menstruación	Edad, duración del ciclo, variación del ciclo, actividad sexual, planes de embarazo, estado de embarazo, IMC, tabaquismo, uso previo de anticonceptivos hormonales	1. Análisis de datos: Ninguno 2. Método de modelización: algoritmo de la tecnología subyacente	Aplicación Natural Cycles 1. Transmisión de datos: Introducción anónima de datos 2. Uso: Identificar el día de la ovulación y el periodo fértil	Detectar la ovulación 1. Retraso medio desde el día de la ovulación 2. Duración de la fase lútea 3. Tasa de falsos positivos
Ecochard et al.^(20)^	2015	Canadá	Estudio observacional	107 mujeres de entre 19 y 45 años, incluidos 326 ciclos	1. TCB: termómetro basal 2. Mucosidad cervical: autoevaluación de la sensación, el aspecto y la consistencia 3. Análisis hormonales: niveles de estrona-3-glucurónido (E1-3-G), pregnanediol-3a-glucurónido (PDG), LH y FSH 4. Estándar de referencia para la ovulación: exploraciones ecográficas	Estrés, enfermedad, insomnio, trastornos del sueño	1. Regla de tres sobre seis 2. Análisis y visualización de datos: software R 3. Método de modelización: algoritmo del último signo fértil	Diagrama 1. Transmisión de datos: Dibujo manual 2. Uso: Identificar la ventana fértil	Ventana fértil Moco cervical 1. Sensibilidad 2. Especificidad
Goodale et al.^(21)^	2019	Suiza	Estudio observacional prospectivo	237 mujeres con una media de 33 años, se incluyeron 708 ciclos menstruales	1. Temperatura nocturna de la piel de la muñeca: pulsera Ava, un dispositivo wearable 2. Parámetros fisiológicos (frecuencia cardíaca, VFC, frecuencia respiratoria y perfusión cutánea): pulsera Ava 3. Calidad del sueño: pulsera Ava 4. Estándar de referencia para la ovulación: ClearBlue Digital Ovulation Test, una prueba de LH en orina para uso doméstico	En las 3 horas previas al sueño, hubo relaciones sexuales, ejercicio intenso, ingesta de alimentos, consumo de café o alcohol	1. Corrección de datos: método Bonferroni 2. Procesamiento y análisis de datos: software R 3. Método de modelización: algoritmo de aprendizaje automático	Aplicación móvil correspondiente 1. Transmisión de datos: sincronización de datos 2. Uso: Detectar el periodo fértil	Ventana fértil Precisión
Scherwitzl et al.^(22)^	2016	Suiza	Estudio retrospectivo	6944 mujeres con una edad media de 29 años, incluidos 272 204 ciclos	1. TCB: termómetro basal 2. Estándar de referencia para la ovulación: pruebas de LH 3. Fecha de la menstruación	Actividad sexual y notas personales	Método de modelización: algoritmo de la tecnología subyacente	Aplicación Natural Cycles 1. Transmisión de datos: Introducir los datos de forma anónima 2. Uso: prevenir el embarazo	1. Tabla de vida de Kaplan-Meier 2. Índice de Pearl de uso perfecto 3. Tasa de fracaso en el uso típico de 13 ciclos 4. Tasa de interrupción típica en 13 ciclos 5. Tasa de interrupción
Demian‘czyk y Michaluk^(23)^	2016	Polonia	Estudio retrospectivo	361 mujeres, incluidos 17 322 ciclos (edad no especificada)	1. TCB: sensor de temperatura 2. Moco cervical: autoevaluación de la sensación 3. Fecha de la menstruación	Método anticonceptivo Uso de preservativos, coitus interruptus, método Creighton, píldora anticonceptiva e, termómetro o dispositivos intrauterinos y observación del moco	1. Procesamiento y análisis de datos: software R 2. Método de modelización: modelos Lady-Comp	Lady-Comp, Pearly y Daysy: dispositivos para controlar el ciclo menstrual 1. Transmisión de datos: introduce los datos de forma anónima 2. Uso: indican las fases fértiles e infértiles del ciclo menstrual para la anticoncepción	Índice Pearl 2010 Índice de Pearl de 2016 Porcentaje de embarazos planificados en mujeres
Manhart y Duane^(24)^		EE. UU.	Estudio prospectivo de cohortes	20 mujeres menores de 40 años, incluidos 240 ciclos	1. TCB: termómetro basal 2. Estándar de referencia para la ovulación: pico de moco (2 días antes y después)	Ninguno	1. Datos faltantes: No se introdujeron los datos faltantes en un ciclo 2. Método de modelización: algoritmo de Natural Cycles	Aplicación Natural Cycles Aplicación CycleProGo 1. Transmisión de datos: introducir los datos de forma anónima 2. Uso: definir el periodo fértil	Equivalente 1. Día de inicio y fin del periodo fértil Aplicación Natural Cycles frente a aplicación CycleProGo 2. Duración media total de la ventana fértil 3. Ciclos con inicio de la ventana fértil 4. Ciclos con finalización de la ventana fértil
Fukaya et al.^(25)^	2017	Japón	Estudio prospectivo de cohortes	20 mujeres, incluidos 27 ciclos (edad no especificada)	1. TCB: termómetro convencional o sensor portátil 2. Fecha de la menstruación Precisión de la predicción secuencial 1. Método convencional de cálculo del calendario 2. Método predictivo secuencial	Ninguna	1. Distribuciones condicionales de la fase: secuencial, técnicas de filtrado bayesiano 2. Datos faltantes: modelo del criterio de información de Akaike (AIC) 3. Método de modelización: modelo de espacio de estados	Sitio web de Ran’s Story, un novedoso marco estadístico e 1. Transmisión de datos: datos introducidos 2. Uso: estimación del ciclo menstrual	Precisión de la predicción Estimación del estado y cálculo de la log-verosimilitud 1. Error cuadrático medio (RMSE) 2. Error absoluto medio (MAE)
Kawamori et al.^(10)^	2019	Japón	Estudio retrospectivo	3533 mujeres de entre 15 y 54 años, incluidos 25 622 ciclos	1. TCB: termómetro convencional o sensor portátil 2. Fecha de la menstruación	Edad Clasificación de cada ciclo menstrual en ocho grupos de edad	1. Distribuciones condicionales de la fase: técnicas de filtrado bayesiano secuencial Identificación de las etapas del ciclo 2. Método de modelización: modelo de espacio de estados autorregresivo con umbral autoexcitado	Sitio web de la historia de Ran 1. Transmisión de datos: introducir datos 2. Uso: estimar el ciclo menstrual	Estimación del estado y cálculo de la log-verosimilitud RMSE
Freundl et al.^(26)^	2014	Alemania	Estudio retrospectivo	51 mujeres de entre 24 y 35 años, incluidos 364 ciclos	1. TCB: termómetro convencional o sensor portátil 2. Fecha de la menstruación	Errores o variaciones en el método de medición, mediciones e es en diferentes momentos, sueño corto o interrumpido, acostarse tarde, cantidades inusuales de alcohol y tensión emocional.	1. Datos faltantes: no se introdujeron los datos faltantes en un ciclo 2. Método de modelización: algoritmo de seguimiento de la señal 3. Método convencional: método sintotérmico de Sensiplan®	Sistema de seguimiento de Trigg (TtS) 1. Transmisión de datos: introducir los datos 2. Uso: definir la ventana fértil	Día de transición TtS y día inicial Sensiplan®
van de Roemer et al.^(27)^	2021	Suiza	Estudio retrospectivo	5328 mujeres con una edad media de 30 años, incluidos 107 020 ciclos	1. TCB: termómetro basal 2. Fecha de la menstruación	Edad, IMC, duración del ciclo, omisión de mediciones, temperatura media alta frente a baja y variaciones de temperatura	1. Datos faltantes: no se introdujeron los datos faltantes en un ciclo 2. Procesamiento y análisis de datos: VE Analyzer 3. Método de modelización: algoritmo Fertility Tracker	Daysy, un dispositivo de seguimiento de la fertilidad 1. Transmisión de datos: introducir los datos 2. Uso: definir la ventana fértil	Análisis de sensibilidad 1. Duración media del ciclo comunicada 2. Tasa de utilización 3. Dispositivo de fertilidad identificado en promedio
Stanford et al.^(28)^	2020	EE. UU.	Estudio prospectivo de cohortes	8363 mujeres de entre 21 y 45 años, incluido 200 712 ciclos	1. TCB: termómetro basal 2. Fluido cervical o posición del cuello uterino: autoevaluación de la sensación 3. Estándar de referencia para la ovulación: prueba de LH en orina	Edad, raza/etnia, embarazos anteriores, IMC, ingresos, tabaquismo actual, educación, educación de la pareja, consumo de cafeína y uso de anticonceptivos hormonales	1. Datos faltantes: utilice la imputación múltiple para imputar los datos faltantes	Aplicaciones móviles: Clue, Fertility Friend, Glow, Kindara y aplicaciones seleccionadas por 1. Transmisión de datos: Encuesta mediante cuestionario 2. Uso: seguimiento del ciclo menstrual y del periodo fértil	1. Tasa de utilización 2. Índices de fecundabilidad (FR) (1) Aumento de la fecundabilidad 3. Tiempo hasta el embarazo (TTP)
Shilaih et al.^(29)^	2018	Suiza	Estudio clínico observacional	136 mujeres de entre 20 y 40 años, incluidos 437 ciclos	1. Temperatura de la piel de la muñeca (WST): pulsera Ava (dispositivo wearable) mientras se duerme 2. Estándar de referencia para la ovulación: ClearBlue Digital Ovulation Test, una prueba de LH en orina para uso doméstico	Consumo de comidas, café, alcohol y relaciones sexuales o ejercicio intenso	Principio: regla de tres sobre seis. 1. Preprocesamiento de datos: LOESS utilizado para suavizar los datos de temperatura 2. Análisis y visualización de datos: software R 3. Método de modelización: modelos lineales de efectos mixtos.	Aplicación Ava, un rastreador de fertilidad informatizado 1. Transmisión de datos: sincronización de datos 2. Uso: Detectar la ovulación	1. Tasa de cambio de temperatura 2. Tiempo de cambio de temperatura 3. Cambio de temperatura
Alzueta et al.^(9)^	2022	EE. UU.	Estudio observacional prospectivo	26 mujeres de entre 18 y 35 años, incluidos 416 ciclos	1. Temperatura distal de la piel durante la noche: Oura Ring, un dispositivo portátil con múltiples sensores 2. Parámetros fisiológicos (FC, VFC y sueño): Oura Ring 3. Calidad del sueño: diario subjetivo 4. Fecha de la menstruación 5. Estándar de referencia para la ovulación: prueba de LH en orina	Calidad del sueño, estado de ánimo, disposición y síntomas físicos	1. Estimación del ciclo menstrual: método Schmalenberger 2. Método de modelización: modelos de regresión lineal jerárquica	Aplicaciones Oura 1. Transmisión de datos: sincronización anónima de datos 2. Uso: estimar el ciclo menstrual	Fases diferentes: Menstruación, ovulación, fase lútea media y fase lútea tardía 1. Temperatura cutánea distal 2. Coeficiente de correlación
Luo et al.^(30)^	2020	China	Estudio prospectivo de cohortes	34 mujeres de entre 22 y 42 años, incluidos 125 ciclos	1. Temperatura interna del oído durante la noche: auricular, un dispositivo intraauricular y no invasivo que se puede llevar puesto 2. TCB: termómetro basal	Enfermedades inflamatorias y situaciones ambientales	1. Preprocesamiento de datos: protocolo de limpieza de datos 2. Algoritmo: un algoritmo de aprendizaje estadístico eficaz y flexible 3. Método de modelización: modelo oculto de Markov (HMM)	Aplicaciones para teléfonos inteligentes 1. Transmisión de datos: Introducir datos 2. Uso: Detectar la ovulación	Auricular frente al método tradicional 1. Precisión de la detección 2. Capacidad de predicción
Maijala et al.^(31)^	2019	Finlandia	Estudio prospectivo de cohortes	22 mujeres de entre 21 y 49 años, incluidos 66 ciclos	1. Temperatura cutánea distal nocturna: Oura Ring, un dispositivo wearable con múltiples sensores 2. TCB: termómetro basal 3. Estándar de referencia para la ovulación: prueba de LH en orina	Calidad del sueño	1. Preprocesamiento de datos: script MATLAB 2. Algoritmo: HALF_LOCS MENSES predice la ovulación para el día intermedio HALF PEAKS predice la ovulación para el primer día	Aplicaciones Oura 1. Transmisión de datos: sincronizar datos 2. Uso: predice los ciclos menstruales y de ovulación	1. Rango de aumento de la temperatura en la fase lútea 2. Correlación entre la temperatura cutánea y la temperatura oral 3. Sensibilidad a la menstruación 4. Duración del periodo fértil
Gombert-Labedens et al.^(32)^	2024	EE. UU.	Estudio observacional prospectivo	120 mujeres de entre 18 y 52 años, incluidos 120 ciclos	1. Temperatura cutánea distal nocturna: Oura Ring, un dispositivo portátil con múltiples sensores 2. Estándar de referencia para la ovulación: prueba de LH en orina	Temperatura y humedad ambientales, tasa metabólica basal, actividad muscular, digestión, sueño, postura y fluctuaciones hormonales	1. Preprocesamiento de datos: script MATLAB 2. Análisis y visualización de datos: software R 3. Método de modelización: Modelos cosinor	Aplicaciones Oura 1. Transmisión de datos: sincronización anónima de datos 2. Uso: estimación del ciclo menstrual	Calidad del ajuste (r2) Datos de temperatura cutánea distal del ciclo menstrual
Hurst et al.^(33)^	2022	EE. UU.	Estudio prospectivo de cohortes	80 mujeres con disfunción ovulatoria e, de entre 22 y 46 años, se incluyeron 205 ciclos	1. Temperatura vaginal nocturna: sensor vaginal (VS) 2. WST nocturna: sensor colocado sobre la piel 3. Estándar de referencia para la ovulación: medición ecográfica de los folículos ováricos y prueba de LH en orina	Ninguno	1. Principio: regla de tres sobre seis. 2. Algoritmo: algoritmo SWS y VS 3. Procesamiento y análisis de datos: método de diferencia de días y método de umbral	Aplicaciones para teléfonos inteligentes 1. Transmisión de datos: sincronización anónima de datos 2. Uso: predecir la ovulación y la concepción	SWS frente a VS 1. Preciso 2. Ventana fértil 3. Tiempo de intento de concepción (TTC)
Regidor et al.^(34)^	2018	Alemania	Estudio prospectivo de cohortes	158 mujeres de entre 18 y 45 años, incluidos 470 ciclos	1. Temperatura corporal central circadiana y circamensual: Ovula Ring, un sensor vaginal 2. Análisis hormonales: niveles de LH, hormona folículoestimulante, estradiol y progesterona 3. Ecografía vaginal	Ninguno	1. Datos expresados en forma de grafico Cyclo Fertilo (CFG). 2. Algoritmo matemático basado en el análisis del ritmo circadian y circamensual de la temperatura corporal central es	Aplicaciones para teléfonos inteligentes 1. Transmisión de datos: sincronización de datos 2. Uso: estimación del ciclo menstrual	1. Error de validación debido a errores de software 2. Precisión en la detección de la ovulación 3. Índice de uso
Wark et al.^(35)^	2015	Australia	Estudio observacional comparativo	16 mujeres de entre 18 y 25 años, incluidos 8 ciclos	1. Temperatura del brazo superior: BodyMedia SenseWear (BMSW) (dispositivo de brazalete) 2. TCB: termómetro digital oral	Ninguno	1. Fecha visual y cuantitativa: MTM 2. Análisis y visualización de datos: gráfico de Bland-Altman (BA)	Aplicación WomanLog Pro 1. Transmisión de datos: Introducir datos 2. Uso: monitorizar la ovulación	1. Rango de temperaturas del termómetro 2. Aceptabilidad de ambos dispositivos

### Características de los Estudios

Los estudios publicados entre 2014 y 2024 se llevaron a cabo en nueve países, seis de ellos en Suiza, cinco en Estados Unidos, dos en China, dos en Japón y dos en Alemania, y uno en Canadá, Polonia, Finlandia y Australia. Las participantes en la investigación eran mujeres sanas en edad fértil de entre 18 y 45 años. Solo un estudio se centró en mujeres con disfunción ovulatoria, a las que se estudió para estimar el ciclo menstrual y detectar la ventana fértil y el día de la ovulación. En cuanto al diseño de la investigación, la mayoría de los estudios fueron prospectivos de cohortes (n = 12, 57,1%) o retrospectivos (n = 6, 28,6%), con una duración de la observación de cuatro a siete ciclos por participante; cinco estudios fueron a gran escala y tres incluyeron encuestas.

### Evaluación de la Calidad

Basándose en la evaluación de la lista de verificación CASP 2024 de los 21 estudios incluidos, esta revisión de alcance identificó una estratificación tripartita en la calidad metodológica. Cinco estudios (23,8%) plantearon preocupaciones importantes, principalmente debido a ajustes incompletos de los factores de confusión (P5–P6), como se observó en Gombert-Labedens et al.^(32)^ y Hurst et al.^(33)^, que carecían de claridad a la hora de abordar las variables, así como en Manhart y Duane^(24)^ y Fukaya et al.^(25)^, que no tuvieron en cuenta estos factores, junto con cuestiones como la falta de claridad de los procesos de reclutamiento (P2) en Hurst et al.^(33)^ o la duración/exhaustividad insuficiente del seguimiento (P7–P8) en Manhart y Duane^(24)^. Tres estudios (14,3%) mostraron preocupaciones menores, entre ellas las relacionadas con la inconsistencia de los métodos de medición de los resultados (P4) en Freundl et al.^(26)^, la presentación parcial de las estimaciones de precisión (P10) en van de Roemer et al.^(27)^ e incertidumbres sobre la aplicabilidad local (P12) en Shilaih et al.^(29)^ y Freundl et al.^(26)^, y en este último y en Regidor et al.^(34)^ se omitió además realizar análisis de estratificación/multivariantes para controlar los factores de confusión. Trece estudios (61,9%) no mostraron ninguna preocupación por el cumplimiento de normas sólidas, en particular en la definición de las preguntas de investigación (P1: cumplimiento del 100%), la medición de los resultados (P4: cumplimiento del 81%) y la presentación de los resultados (P9–P11: cumplimiento del 76–100%). Sin embargo, persistieron deficiencias generalizadas, cuantificadas en la Tabla 3 como cumplimiento subóptimo del control de factores de confusión (P5–P6: 61,9%, con 8 estudios deficientes), limitaciones en la exhaustividad del seguimiento (P7–P8: 71,4% de cumplimiento) y validación insuficiente de la aplicabilidad local (P12: 47,6% de cumplimiento), lo que en conjunto subraya la necesidad de que las investigaciones futuras den prioridad a los ajustes preespecificados de los factores de confusión, al seguimiento prolongado ≥ 6 ciclos menstruales y al muestreo diversificado de la población para mejorar la fiabilidad de la evidencia y la utilidad clínica. La evaluación general de cada estudio se presenta en la Tabla 3.

**Tabla 3:** Lista de verificación CASP Resumen de los estudios incluidos.

	Zhu et al.^(8)^	Yu et al.^(18)^	Scherwitzl et al.^(19)^	Ecochard et al.^(20)^	Goodale et al.^(21)^	Scherwitzl et al.^(22)^	Demianczyk y Michaluk^(23)^
P1	Sí	Sí	Sí	Sí	Sí	Sí	Sí
P2	Sí	Sí	No sé con seguridad con seguridad	Sí	Sí	No	No sé con seguridad
P3	Sí	Sí	Sí	Sí	Sí	No sé con seguridad	Sí
P4	Sí	Sí	Sí	Sí	Sí	No	Sí
P5	Sí	Sí	No	No	Sí	No	No
P6	No	Sí	No	No	Sí	No	No
P7	No sé con seguridad	Sí	No	No	Sí	No	Sí
P8	Sí	Sí	No	Sí	Sí	No	Sí
P9	Sí	Sí	Sí	Sí	Sí	Sí	Sí
P10	Sí	Sí	No sé con seguridad	Sí	Sí	Sí	No sé con seguridad
P11	Sí	Sí	Sí	Sí	Sí	No sé con seguridad	Sí
P12	No	No sé con seguridad	No sé con seguridad	Sí	No sé con seguridad	No	No sé con seguridad
P13	Sí	Sí	Sí	Sí	Sí	Sí	Sí
P14	Sí	Sí	Sí	Sí	Sí	No sé con seguridad	Sí
	**Manhart y Duane** ^(24)^	**Fukaya et al.** ^(25)^	**Kawamor** ^(10)^	**Freundl et al.** ^(26)^	**van de Roemer et al.** ^(27)^	**Stanford et al.** ^(28)^	**Shilaih et al.** ^(29)^
P1	Sí	Sí	Sí	Sí	Sí	Sí	Sí
P2	Sí	Sí	No sé con seguridad	Sí	Sí	Sí	Sí
P3	No sé con seguridad	No sé con seguridad	Sí	Sí	Sí	No sé con seguridad	Sí
P4	Sí	Sí	Sí	No sé con seguridad	Sí	Sí	Sí
P5	No	No	No	No	No	Sí	Sí
P6	No	No	No sé con seguridad	No	No	Sí	Sí
P7	Sí	Sí	No sé con seguridad	Sí	Sí	Sí	No sé con seguridad
P8	Sí	Sí	Sí	Sí	Sí	Sí	Sí
P9	Sí	Sí	Sí	Sí	Sí	Sí	Sí
P10	Sí	Sí	Sí	Sí	Sí	Sí	Sí
P11	Sí	Sí	Sí	Sí	Sí	Sí	Sí
P12	No sé con seguridad	No	No sé con seguridad	No sé con seguridad	No sé con seguridad	No	Sí
P13	Sí	No sé con seguridad	Sí	Sí	Sí	Sí	Sí
P14	Sí	Sí	Sí	Sí	Sí	Sí	Sí
	**Alzueta et al.** ^(9)^	**Luo et al.** ^(30)^	**Maijala et al.** ^(31)^	**Gombert-Labedens et al.** ^(32)^	**Hurst et al.** ^(33)^	**Regidor et al.** ^(34)^	**Wark et al.** ^(35)^
P1	Sí	Sí	Sí	Sí	Sí	Sí	Sí
P2	Sí	No sé con seguridad	No sé con seguridad	Sí	No	No sé con seguridad	No sé con seguridad
P3	Sí	Sí	Sí	Sí	Sí	Sí	Sí
P4	Sí	No sé con seguridad	Sí	No sé con seguridad	Sí	Sí	Sí
P5	No	No	Sí	No	Sí	No	No
P6	No sé con seguridad	Sí	No sé con seguridad	No	Sí	No	No
P7	Sí	Sí	Sí	Sí	No	Sí	Sí
P8	Sí	Sí	Sí	No	Sí	Sí	Sí
P9	Sí	Sí	Sí	Sí	Sí	Sí	Sí
P10	Sí	Sí	Sí	No sé con seguridad	Sí	No sé con seguridad	No sé con seguridad
P11	Sí	No sé con seguridad	Sí	Sí	Sí	Sí	Sí
P12	Sí	No sé con seguridad	No sé con seguridad	No sé con seguridad	No sé con seguridad	No sé con seguridad	No sé con seguridad
P13	Sí	Sí	Sí	Sí	Sí	Sí	Sí
P14	Sí	Sí	Sí	Sí	Sí	Sí	Sí

1. ¿El estudio abordó un tema claramente definido?

2. ¿Se reclutó la cohorte de forma aceptable?

3. ¿Se midió la exposición con precisión para minimizar el sesgo?

4. ¿Se midieron los resultados con precisión para minimizar el sesgo?

5. ¿Han identificado los autores todos los factores de confusión importantes?

6. ¿Se han tenido en cuenta los factores de confusión en el diseño y/o el análisis?

7. ¿El seguimiento de los sujetos fue lo suficientemente completo?

8. ¿El seguimiento de los sujetos fue lo suficientemente prolongado?

9. ¿Cuáles son los resultados de este estudio?

10. ¿Qué grado de precisión tienen los resultados?

11. ¿Cree usted en los resultados?

12. ¿Se pueden aplicar los resultados a la población local?

13. ¿Los resultados de este estudio concuerdan con otras pruebas disponibles?

14. ¿Qué implicaciones tiene este estudio para la práctica?

### Contenido y Métodos de Medición

Nueve estudios analizaron el uso de dispositivos portátiles, entre ellos una pulsera, un anillo, dispositivos vaginales, auriculares y brazaletes, para medir la temperatura corporal (Tabla 4). Estos novedosos dispositivos portátiles miden de forma continua y automática la temperatura de la piel durante el sueño para definir la TCB, y los datos se recogen en la fase media de la noche para evitar las perturbaciones de las fases de conciliación del sueño y despertar^(8)^. Además, 16 estudios tuvieron en cuenta algunos factores de confusión que influyen en la TCC, entre ellos factores individuales (por ejemplo, edad, peso, enfermedades y medicamentos), factores psicosociales (por ejemplo, estrés, insomnio y cambio climático) y factores relacionados con el estilo de vida (por ejemplo, actividad sexual, tabaquismo y consumo de alcohol y café). Además, Yu et al.^(18)^ y Alzueta et al.^(9)^ utilizaron dispositivos portátiles multisensor (la Huawei Band y el Oura Ring) para medir parámetros fisiológicos (frecuencia cardíaca [FC], variabilidad de la FC [VFC] y sueño) con el fin de mejorar la precisión de la predicción. Otros estudios utilizaron un termómetro basal para analizar cómo mejorar la precisión de la predicción mediante la actualización del algoritmo.

**Table 4:** Características y funciones de los dispositivos portátiles.

Tipo de dispositivo	Dispositivo de pulsera	Dispositivo anillo	Dispositivo vaginal	Dispositivo para el oído	Dispositivo para el brazo
P Nombre del producto e	Pulsera Ava	Anillo Oura	Ovula	Auricular	BodyMedia SenseWear
Medición	Temperatura de la piel de la muñeca (WST)	Temperatura de la piel distal (DST)	Temperatura corporal central (CBT)	Temperatura interna de la oreja (EIT)	Temperatura en la parte superior del brazo (UAT)
Características	1. Frecuencia de registro: cada 10 s durante toda la noche 2. Al menos 4 horas de sueño ininterrumpido	1. Frecuencia de registro: cada 30 s durante toda la noche 2. Cada 30 s de monitorización del sueño durante toda la noche	1. Frecuencia de registro: cada 5 minutos durante todo el ciclo 2. Medición intravaginal exacta circadiana y circamensual	1. Frecuencia de registro: cada 5 minutos durante toda la noche 2. Eliminar al menos los primeros 20–30 minutos de datos para estabilizar	1. Frecuencia de registro: cada 5 minutos durante toda la noche 2. Borrar 60 minutos de datos antes de despertarse
Contenido de la investigación	1. Predicción del periodo fértil 2. Seguimiento del ciclo menstrual	Seguimiento del ciclo menstrual	Predicción de la ventana fértil y la ovulación	Predicción de la ovulación	Predicción de la ovulación

### Experiencia del Usuario con Dispositivos Portátiles

En el panorama tecnológico actual de los dispositivos portátiles para el control de la fertilidad, cinco categorías (pulseras, anillos, sensores vaginales, dispositivos para el oído y brazaletes) muestran claras diferencias en cuanto a rendimiento y adaptabilidad a cada usuaria. Las pulseras permiten un seguimiento continuo y discreto mediante la monitorización de la temperatura de la piel de la muñeca, con una gran resistencia a las interferencias ambientales, pero requieren una recarga diaria y pueden limitar el movimiento de la muñeca. No obstante, son adecuadas para mujeres que desean quedarse embarazadas y dan prioridad a la comodidad, aunque la incomodidad relacionada con el sueño puede comprometer su cumplimiento^(8,21,29)^. Con capacidad impermeable (hasta 50 metros de resistencia al agua y compatibles con la sauna), alta integridad de los datos (>97%) y una duración de la batería de 3 días, los anillos se adaptan a las mujeres con horarios irregulares, pero las estrictas restricciones de tamaño y la dependencia de la sincronización manual suponen un riesgo de descuidos operativos^(9,31,32)^. Los sensores vaginales proporcionan mediciones precisas de la temperatura corporal central sin necesidad de tomar muestras de orina, lo que los hace ideales para personas que cruzan husos horarios o trabajan por turnos, pero su diseño invasivo, asociado a molestias menstruales y barreras psicológicas, y la necesidad de sustituirlos mensualmente limitan su uso principalmente a pacientes e es con infertilidad que requieren datos de alta precisión^(34)^. Los dispositivos que se llevan en la oreja atraen a los usuarios expertos en tecnología gracias a la monitorización estable de la temperatura del conducto auditivo y al análisis totalmente automatizado mediante inteligencia artificial, aunque su tendencia a desprenderse durante el sueño y el riesgo de irritación del conducto auditivo pueden provocar lagunas en los datos nocturnos^(18,30)^. Con una duración de la batería de 7 días, una irritación mínima de la piel y una monitorización multiparamétrica, las bandas para el brazo son útiles en ensayos clínicos, pero se enfrentan a limitaciones prácticas debido a una tasa de pérdida de mediciones del 35% y a la necesidad de contar con expertos para interpretar los datos^(35)^.

En general, la selección del dispositivo requiere equilibrar la precisión (óptima en los sensores vaginales) y el cumplimiento (mejor en las pulseras y los anillos), junto con consideraciones de aceptabilidad psicológica (mayor en los dispositivos no invasivos) y exigencias específicas de cada situación (la monitorización de grado médico a menudo sacrifica la comodidad). Las investigaciones futuras deben dar prioridad a las optimizaciones técnicas (por ejemplo, la prolongación de la duración de la batería y la reducción de la invasividad) y a las adaptaciones personalizadas (basadas en la tolerancia ocupacional o fisiológica) para mejorar la fiabilidad a largo plazo y la adherencia de los usuarios. Estas consideraciones para los dispositivos portátiles se presentan en la Tabla 5.

**Tabla 5:** Consideraciones sobre la experiencia del usuario para los dispositivos portátiles.

Tipo de dispositivo	Ventajas	Desventajas	Usuarios objetivo	Experiencias negativas
Pulsera	1. Monitorización continua discreta (temperatura de la piel de la muñeca) 2. Gran resistencia a las interferencias ambientales 3. Sincronización automática de datos con la aplicación	1. Requiere recarga diaria 2. Posibles restricciones en el movimiento de la muñeca	Mujeres que buscan un seguimiento cómodo de la fertilidad	Molestias al llevarlo puesto durante el sueño
Anillo	1. Resistente al agua (50 m de profundidad) y apto para sauna 2. Disponibilidad de datos de > 97% 3. Duración de la batería de 3 días	1. Se requiere sincronización manual diaria de los datos 2. Requisitos estrictos en cuanto al tamaño de los dedos	Mujeres con trabajos con horarios irregulares	Riesgo de olvidar las rutinas de sincronización
Vaginal	1. Medición más precisa de la temperatura corporal 2. No es necesario recoger muestras de orina 3. Adaptable a trabajadores por turnos/viajeros con cambio de huso horario	1. Posibles molestias menstruales 2. Requiere sustitución mensual 3. Resistencia psicológica	Pacientes con infertilidad que necesitan precisión	Barreras psicológicas para la inserción
Oído	1. Alta estabilidad de la temperatura del canal auditivo 2. Monitorización totalmente automatizada 3. Análisis mediante aplicación con inteligencia artificial	1. Fácil extracción durante el sueño 2. Riesgo de irritación para usuarios sensibles	Usuarios pioneros en la adopción de nuevas tecnologías	Lagunas en los datos debido al movimiento nocturno
Brazalete	1. Batería de larga duración (7 días) 2. Baja irritación cutánea 3. Monitorización multiparamétrica	1. Bajo cumplimiento (35% de mediciones perdidas) 2. Requiere interpretación de datos por parte de expertos	Participantes en ensayos clínicos	Percepción de movimiento restringido del brazo

### Análisis de Monitoreo a Largo plazo e Implicaciones Clínicas

La literatura existente indica que el monitoreo de la temperatura mediante tecnología portátil presenta ventajas considerables y oportunidades de investigación para el manejo de los ciclos menstruales durante períodos prolongados. La pulsera Ava, en particular, demostró fluctuaciones significativas en la temperatura de la piel de la muñeca (WST), la frecuencia cardíaca y la frecuencia respiratoria durante un año de monitoreo continuo. Esta investigación confirmó que los algoritmos de aprendizaje automático pueden alcanzar una tasa de precisión del 90% en la predicción de la ventana fértil mediante la síntesis de datos multiparamétricos, lo que destaca la importancia de los datos a largo plazo para la optimización de estos algoritmos^(21)^.

El estudio que examinó el dispositivo Daysy destacó la importancia fundamental del cumplimiento por parte de las usuarias; concretamente, cuando la frecuencia de las mediciones superaba el 80%, la precisión de los resultados del algoritmo mejoraba significativamente, con un 42,4% de «días verdes» identificados con precisión dentro de los periodos fértiles. Además, se mantuvo la estabilidad de la duración de la fase lútea (media de 12,7 ± 1,4 días), lo que subraya la necesidad de un monitoreo constante para la eficacia de los métodos anticonceptivos naturales^(27)^. El monitoreo con WST validó aún más su potencial como alternativa a la medición oral de la TCB al mostrar un patrón bifásico que se correlacionaba con la TCB oral (r = 0,563) y una elevación de 0,30 °C durante la fase lútea en comparación con las temperaturas de la fase folicular, aunque persistieron los retos relacionados con la interferencia ambiental^(29)^. Además, la aplicación del modelo coseno a los datos de la TCB a largo plazo identificó con éxito el 82% de los ciclos bifásicos mediante la cuantificación de características rítmicas como la mediana, la amplitud y la fase pico, al tiempo que señaló los ciclos anormales a través de desviaciones en estos parámetros, proporcionando así una herramienta cuantitativa para evaluar la salud menstrual^(32)^. En resumen, el monitoreo a largo plazo no solo aclara la estabilidad dinámica de los parámetros fisiológicos, como la consistencia de la temperatura y la duración de la fase lútea, sino que también revela limitaciones técnicas, incluida la variabilidad de los dispositivos y los factores de confusión ambientales.

### Procesamiento y Análisis de Datos Complejos

Nueve estudios recolectaron datos simultáneamente a través de la sincronización Bluetooth, mientras que los demás se basaron en la entrada manual de datos por parte de las participantes, lo que pudo haber dado lugar a errores de introducción. Para proteger la privacidad de las pacientes y ocultar las diferencias entre subgrupos, los datos se recolectaron de forma anónima en siete estudios. Debido a la naturaleza densa y compleja de los datos de temperatura, algunos estudios realizaron un preprocesamiento de los datos. Por ejemplo, de acuerdo con un protocolo de limpieza de datos, estos se limpiaron y organizaron para garantizar su precisión, consistencia y exhaustividad^(30)^, y se desarrolló un script MATLAB para gestionarlos^(31)^. Además, cada parámetro se sometió a un suavizado de diagramas de dispersión estimado localmente (locally estimated scatterplot smoothing – LOESS) antes del análisis estadístico^(19)^. En cuanto a los datos faltantes, cinco estudios los excluyeron y un estudio utilizó imputaciones múltiples para imputar los datos faltantes^(28)^. En cuanto al análisis de los datos, algunas investigaciones presentaron los datos en forma de gráficos y tablas para analizarlos mejor^(34,35)^. Cuatro estudios explicaron cómo la TCB predecía la ovulación basándose en la regla de «tres sobre seis», que significa que se requieren tres temperaturas 0,2 °F por encima del punto más alto de las seis temperaturas anteriores, con al menos una de las temperaturas más altas 0,4 °F por encima de las más bajas (2). Freundl et al.^(26)^ utilizó los métodos sintotérmicos Sensiplan® para enriquecer aún más la regla. Además, el 33,3% de los estudios utilizaron el software R para el análisis y la visualización de los datos, el 23,8% construyeron modelos lineales de efectos mixtos y el 76,2% adoptaron algoritmos y modelos innovadores para la predicción. Es necesario seguir investigando para mejorar la precisión de las predicciones.

### Funcionalidad y Eficacia de las Aplicaciones

Las principales funciones de las aplicaciones incluyen cuatro aspectos: estimación del ciclo menstrual (8 aplicaciones), predicción de la fertilidad (14 aplicaciones), indicación de anticonceptivos (2 aplicaciones) y monitoreo del embarazo (1 aplicación). Entre las aplicaciones disponibles actualmente se encuentran Ava, Natural Cycles y Oura, WomanLog Pro, así como el sitio web Ran’s Story y los ordenadores Pearly y Daysy Cycle. Stanford et al.^(28)^ evaluó la fecundabilidad de cinco aplicaciones (Clue, Fertility Friend, Glow, Kindara y Ovia) y las consideró eficaces, pero los resultados carecían de pruebas. Los parámetros de evaluación de la eficacia de las aplicaciones fueron el cambio de temperatura, la duración de la ventana fértil y el rendimiento del modelo, incluyendo la precisión, la sensibilidad, la especificidad, el error cuadrático medio (RMSE), el error absoluto medio (MAE), el coeficiente de correlación, el índice de fertilidad (índice de Pearl, índices de fecundidad y tiempo hasta el embarazo) y la disponibilidad de la aplicación (tasas de utilización y abandono).

## DISCUSIÓN

La mayoría de los estudios examinados en esta revisión demostraron que la TCB puede predecir la ovulación, y la TCB está reconocida como un indicador de fertilidad^(36)^. Sin embargo, la medición tradicional de la TCB requiere un horario y un lugar fijos, normalmente cada mañana antes de levantarse, lo que implica introducir manualmente los datos en una aplicación y medir la temperatura corporal para realizar un seguimiento de la menstruación y la ovulación^(37,38)^. Esta rutina diaria puede ser tediosa y estar sujeta a errores de notificación^(36,39)^. En esta revisión de alcance, se encontró que el 42% de la investigación se centraba en el uso de dispositivos portátiles para predecir los ciclos menstruales y la ovulación, impulsada por los avances en sensores portátiles y tecnología portátil que permiten la recolección continua y dinámica de información de salud a lo largo del día^(40,41)^. Los resultados de este estudio confirman que los dispositivos portátiles que miden continuamente la temperatura de la piel y sincronizan automáticamente los datos parecen ser muy adecuados para abordar las limitaciones actuales de las aplicaciones de seguimiento tradicionales y basadas en la TCB^(22)^. Estos dispositivos pueden llevarse en diversas partes del cuerpo —la muñeca, el dedo, la parte superior del brazo, el interior de la vagina o el oído— y permiten un seguimiento longitudinal más exhaustivo de los parámetros fisiológicos, lo que permite a las usuarias observar patrones personalizados en los datos evolutivos^(22,42)^. Además, estos dispositivos también pueden evaluar el estado físico general midiendo cambios fisiológicos, como cambios en la FC, la VFC, la frecuencia respiratoria, la perfusión cutánea y la calidad del sueño^(9,19,22)^. En general, en comparación con los dispositivos convencionales, los dispositivos portátiles tienen el potencial de mejorar el seguimiento de la TCB mediante la medición continua de múltiples parámetros fisiológicos, lo que conduce a una estimación más precisa de la ovulación. Se necesitan más investigaciones para validar estos avances. Las principales conclusiones y aplicaciones clínicas por tipo de dispositivo se enumeran en la Tabla 6.

**Tabla 6:** Importancia clínica de los diferentes tipos de dispositivos.

Tipo de dispositivo	Importancia clínica	Conclusiones principales
Pulsera	1. Seguimiento continuo y discreto de las tendencias ovulatorias de la TCB 2. El diseño anti-interferencias mejora la fiabilidad de la evaluación básica de la fertilidad	**Ventajas:** Su aplicación universal para el control diario de la fertilidad prioriza la comodidad **Desventajas:** Las molestias durante el sueño provocan un 23% de abandono. **Indicación:** Herramienta de cribado primario para la población general que intenta concebir
Anillo	1. El diseño resistente al agua permite capturar datos en entornos especiales (por ejemplo, al nadar o en la sauna) 2. La alta integridad de los datos mejora la precisión de la predicción de ciclos	**Ventajas:** La batería de larga duración y las características de resistencia al agua son ideales para grupos profesionales que trabajan en condiciones muy exigentes **Contras:** Las limitaciones de tamaño excluyen al 5–15% de las mujeres (circunferencia del dedo inadecuada) **Indicación:** Monitoreo a largo plazo para poblaciones con horarios irregulares
Vaginal	1. Precisión de la temperatura corporal central (±0,05 °C, nivel de referencia) 2. Elimina los errores de muestreo de la orina matutina	**Ventajas:** Proporciona datos de calidad médica para intervenciones de tecnología de reproducción asistida **Desventajas:** > 40% de abandono debido a barreras psicológicas, especialmente para los usuarios primerizos **Indicaciones:** Esencial para el monitoreo preciso en ciclos de ART
Oído	1. La estabilidad de la temperatura del canal auditivo supera la de la superficie cutánea en 0,1–0,3 °C Los algoritmos de IA aumentan las tasas de detección de ovulaciones anormales	**Ventajas:** El análisis automatizado reduce la carga cognitiva del usuario. **Desventajas:** El desplazamiento nocturno provoca una pérdida de datos críticos (~30%) **Indicación:** Gestión personalizada de la fertilidad para usuarios familiarizados con la tecnología
Brazalete	1. Análisis multiparamétrico (temperatura + variabilidad de la frecuencia cardíaca) 2. La batería de larga duración permite investigar los trastornos crónicos de la fertilidad	**Ventajas:** Biocompatibilidad cutánea superior (< 2% de hipersensibilidad) **Desventajas:** Las altas tasas de pérdida de datos limitan su utilidad clínica **Indicaciones:** Exploración de mecanismos fisiológicos en entornos de investigación

La fusión de la tecnología de sensores portátiles con algoritmos de aprendizaje automático ha avanzado significativamente el desarrollo de aplicaciones inteligentes de seguimiento de la fertilidad^(43)^. Esta integración tiene el potencial de permitir una caracterización completa de la paciente y optimizar las intervenciones clínicas^(44)^. Para hacer realidad esta visión, es fundamental realizar una estimación precisa de los datos de series temporales de la TCB, y varios estudiosos han intentado subsanar cualquier deficiencia. Por ejemplo, Fukaya et al.^(25)^ y Kawamori et al.^(10)^ desarrollaron un modelo de espacio de estados que incluye la fase menstrual como variable de estado latente para explicar las fluctuaciones diarias de la TCB y la duración del ciclo menstrual. El modelo de espacio de estados se basa en técnicas de filtrado bayesiano secuencial para mapear los datos en un espacio de estados con el fin de capturar las relaciones de dependencia a largo plazo, y se emplea comúnmente para describir y analizar el comportamiento de sistemas dinámicos en la investigación académica^(45)^. De manera similar, Luo et al.^(30)^ utilizó un modelo de Markov oculto para describir la relación probabilística entre la observación y las secuencias de estados ocultos con el fin de predecir los resultados de observaciones futuras o clasificar una secuencia según el proceso oculto potencial de los datos generados. Para analizar más a fondo el ritmo circadiano de la temperatura de la piel, algunos estudios aplicaron el modelo cosinor para facilitar la evaluación de los efectos del ciclo menstrual en los parámetros fisiológicos y en entornos clínicos, utilizando las características de los ciclos menstruales como marcadores de salud o para facilitar la cronoterapia menstrual^(46)^. Algunos presentaron los resultados del análisis de datos en un formato visual, lo que ayudó a los usuarios a comprender mejor los hallazgos^(22)^. El algoritmo tecnológico subyacente se basaba en los datos introducidos cada día, y los resultados se presentaban como un icono rojo (riesgo) o verde (sin riesgo) para indicar el riesgo de embarazo^(22)^. Los algoritmos de aprendizaje automático seguirán ofreciendo más posibilidades para mejorar la precisión de las aplicaciones de seguimiento de la fertilidad en el futuro.

Una encuesta piloto realizada en Internet reveló que aproximadamente una cuarta parte de las personas encuestadas afirmaban utilizar aplicaciones de seguimiento de la fertilidad, y el 63% de los usuarios coincidían en que las aplicaciones tenían una base científica y eran eficaces para determinar el periodo fértil^(47)^. Sin embargo, la sensibilidad de las aplicaciones de seguimiento de la fertilidad varía mucho, desde el 62% de los sensores que se llevan en la muñeca hasta el 28% de los dispositivos que se llevan en la oreja^(30,48)^. Moglia et al.^(49)^ utilizó el sistema de puntuación “APPLICATIONS” para evaluar las aplicaciones de seguimiento del ciclo menstrual y descubrió que la mayoría de las aplicaciones gratuitas para teléfonos inteligentes eran inexactas^(50)^. Muchas aplicaciones relacionadas con la fertilidad pueden ser menos precisas y menos propensas a publicar sus datos en revistas revisadas por pares. Por lo tanto, la dependencia de estas aplicaciones puede dar lugar a embarazos no deseados o a retrasos en la identificación de problemas de infertilidad, lo que puede tener consecuencias indelebles^(7,39,47)^. Un análisis más detallado de la experiencia de las usuarias también reveló que estas tenían preocupaciones sobre la inexactitud de las fechas previstas, el costo y la privacidad de los datos, así como ansiedad y frustración por la falta de fiabilidad de las predicciones del ciclo menstrual^(8,51)^. Dado que se observó que el uso de las aplicaciones disminuía gradualmente con el tiempo, vale la pena estudiar cómo mejorar la adherencia y la precisión de las predicciones.

Las mujeres que utilizan estas aplicaciones esperan recibir información científica y completa sobre salud reproductiva, así como apoyo emocional y asesoramiento sobre decisiones anticonceptivas, salud sexual y cuidados posoperatorios^(47)^. Sin embargo, una revisión reveló que no todas las aplicaciones disponibles se basan en pruebas^(37)^ y que solo el 17% proporciona información sobre anticoncepción^(49)^. Por lo tanto, los diseñadores de aplicaciones deben tener en cuenta las necesidades diversas y cambiantes de las usuarias, atendiendo a una gama más amplia de propósitos, como la anticoncepción y el seguimiento de la fertilidad, al tiempo que observan la respuesta a la medicación, monitorean las enfermedades reproductivas y llegan a un público más diverso^(5,7)^. Para obtener información más precisa sobre la fertilidad, es fundamental proporcionar formación preliminar sobre los métodos de medición y los pasos para utilizar la aplicación. Por ejemplo, se puede obtener formación personalizada sobre el método sintotérmico a través de la International Couple to Couple League (https://ccli.org/)^(52)^.

## LIMITACIONES DEL ESTUDIO

Aunque prometedor, el conjunto de pruebas actuales sobre la tecnología de seguimiento de la fertilidad basada en la TCB presenta una heterogeneidad significativa y limitaciones metodológicas que requieren una interpretación cautelosa. Tal y como se ha identificado en la evaluación de calidad del CASP, entre las principales preocupaciones que afectan al rigor y la comparabilidad de los resultados se encuentra el hecho de que una proporción considerable de los estudios no abordaron o informaron adecuadamente sobre los factores de confusión que se sabe que influyen en la TCB (por ejemplo, enfermedades, medicación, cambios significativos en el estilo de vida y trastornos del sueño). Esta omisión introduce un sesgo y limita la capacidad de aislar los efectos reales de la tecnología de seguimiento en sí misma sobre resultados como la precisión de la predicción de la ovulación y la eficacia anticonceptiva. Otra limitación fue que el estudio incluyó poblaciones diversas de diferentes países (por ejemplo, Suiza, Estados Unidos, China y Japón) con rangos de edad variables, tanto sanas como con problemas de salud (por ejemplo, disfunción ovulatoria). Aunque esto refleja la diversidad del mundo real, también contribuye a la heterogeneidad de los resultados. El estado de fertilidad inicial, la regularidad del ciclo, los factores culturales que influyen en el cumplimiento y el acceso a la tecnología variaron considerablemente. Una tercera limitación fue que los estudios utilizaron diferentes dispositivos portátiles (pulseras, anillos, sensores vaginales y auriculares) y termómetros tradicionales, cada uno con una variabilidad de medición inherente. Es fundamental señalar que los estándares de referencia para confirmar la ovulación también difirieron (pruebas de hormona luteínica en orina, ecografía, nivel de hormonas en suero y evaluación del moco cervical), cada uno con una sensibilidad y especificidad variables. Esta heterogeneidad dificulta la comparación directa del rendimiento de la aplicación y del dispositivo entre los distintos estudios. Por último, aunque el uso de cohortes prospectivas era habitual, varios estudios clave se basaban en diseños retrospectivos o carecían de una duración o exhaustividad suficientes, lo que suscitaba preocupaciones sobre el sesgo de selección y el sesgo de desgaste, y solo se realizaron cinco estudios a gran escala. Estas limitaciones ponen de relieve la necesidad de realizar estudios prospectivos de alta calidad que utilicen protocolos estandarizados para controlar los factores de confusión, al tiempo que se emplean estándares de referencia sólidos (como ecografías seriadas y mediciones de progesterona sérica), se informan claramente las metodologías de los algoritmos (o se hacen de código abierto) y se incluyen poblaciones diversas representativas de los usuarios previstos.

## CONCLUSIÓN

Esta revisión reveló varias áreas que requieren más investigación y sobre las que se pueden formular recomendaciones. En primer lugar, los dispositivos portátiles deben recolectar datos de manera que se evite la confusión con otros factores y se mejore aún más la precisión de los datos. En segundo lugar, se deben desarrollar algoritmos innovadores de aprendizaje automático para procesar grandes muestras de datos de series temporales y explorar el mejor algoritmo. En tercer lugar, se debe proporcionar información personalizada, completa y científica sobre la fertilidad a las mujeres que intentan concebir o prevenir el embarazo.

En cuarto lugar, se debe prestar especial atención a la experiencia del usuario, la interacción y la privacidad. En quinto lugar, se debe mejorar el sistema de formación y evaluación de las aplicaciones de seguimiento de la fertilidad. Es necesario realizar más investigaciones para aplicar estas recomendaciones y mejorar el valor del seguimiento de la fertilidad basado en la TCB, al tiempo que se promueve su desarrollo.

## Data Availability

Todo el conjunto de datos que respalda los resultados de este estudio se publicó en el artículo y en la sección de “Materiales Complementarios”.
